# Translation of yes-associated protein (YAP) was antagonized by its circular RNA via suppressing the assembly of the translation initiation machinery

**DOI:** 10.1038/s41418-019-0337-2

**Published:** 2019-05-15

**Authors:** Nan Wu, Zhidong Yuan, Kevin Y. Du, Ling Fang, Juanjuan Lyu, Chao Zhang, Alina He, Esra Eshaghi, Kaixuan Zeng, Jian Ma, William W. Du, Burton B. Yang

**Affiliations:** 1 Sunnybrook Research Institute, S-Wing Research Building, 2075 Bayview Ave, Toronto, M4N 3M5 Canada; 2 Department of Laboratory Medicine and Pathobiology, University of Toronto, Toronto, Canada; 3 School of Basic Medicine, Gannan Medical University, Ganzhou, Jiangxi China; 4 China-Japan Union Hospital of Jilin University, Jilin, China

**Keywords:** RNA, Gene expression

## Abstract

Yap is the key component of Hippo pathway which plays crucial roles in tumorigenesis. Inhibition of Yap activity could promote apoptosis, suppress proliferation, and restrain metastasis of cancer cells. However, how Yap is regulated is not fully understood. Here, we reported Yap being negatively regulated by its circular RNA (circYap) through the suppression of the assembly of Yap translation initiation machinery. Overexpression of circYap in cancer cells significantly decreased Yap protein but did not affect its mRNA levels. As a consequence, it remarkably suppressed proliferation, migration and colony formation of the cells. We found that circYap could bind with Yap mRNA and the translation initiation associated proteins, eIF4G and PABP. The complex containing overexpressed circYap abolished the interaction of PABP on the poly(A) tail with eIF4G on the 5′-cap of the Yap mRNA, which functionally led to the suppression of Yap translation initiation. Individually blocking the binding sites of circYap on Yap mRNA or respectively mutating the binding sites for PABP and eIF4G derepressed Yap translation. Significantly, breast cancer tissue from patients in the study manifested dysregulation of circYap expression. Collectively, our study uncovered a novel molecular mechanism in the regulation of Yap and implicated a new function of circular RNA, supporting the pursuit of circYap as a potential tool for future cancer intervention.

## Introduction

Yes-associated protein (Yap) is the most essential member of Hippo pathway [1, 2]. The transcriptional coactivator Yap shuttles from cytoplasm to nucleus, interacts with TEA domain family members (TEAD) and thus promotes the transcription of a variety of oncogenes [3, 4]. Therefore, activation of Yap incites the proliferation, restrains apoptosis, and promotes metastasis of cancer cells [5–7]. To date, Hippo pathway is the best understood mechanism to restrict the oncogenic properties of Yap [2, 8]. Recently, new mechanisms for mechanoregulation of Yap and Yap-mediated cancer cell transcriptional addiction were unveiled, which further emphasized the essential role of Yap in tumorigenesis [9, 10]. However, most of these regulators target phosphorylation and translocation of Yap, and these are usually mediated by other proteins.

Translational control is a crucial component of cancer development and progression, directing both global control of protein synthesis and selective translation of mRNAs that promote tumor cell survival [11]. Classically, the translation initiated from the circularization of mRNA and the binding of PABP on the poly(A) tail and eIF4G on the 5′-cap of the mRNA translation initiation complex. Blocking the interaction of eIF4G and PABP directly prevents the start of translation, and therefore suppresses the synthesis of protein.

Circular RNA (circRNA) is a group of transcripts in which the 3′ and 5′ ends covalently joined [12, 13]. Recently circRNA were found to be involved in physiological and pathological procedures as these have the potential to promote or suppress cancer development and regulates neural activities [14–17]. The first mechanism that was identified with regards to the role of circRNA was microRNA sponge [18]. In addition, circRNA could also bind with proteins in the related signal pathways [19–22]. While circRNAs can be translated to protein peptides [23, 24], it is not known whether circRNA could regulate protein translation, especially the protein from their parental RNA. Our present study was designed to discover a new regulation mechanism for Yap during tumorigenesis by investigating whether Yap circular RNA (circYap) is able to directly and selectively regulate Yap translation.

## Results

### Yap protein expression was antagonized by circYap

**Fig. 1:**
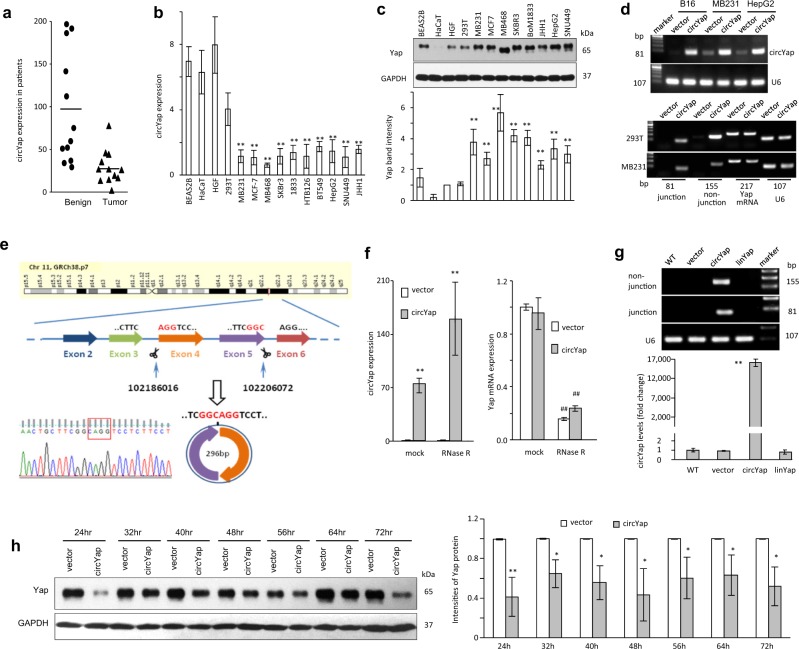
The circYap expression in tissues and cells. **a** The expression of circYap in the tumor and paracancerous tissues of breast cancer patients. *n* = 12. **b** The expression of circYap in immortalized non-cancerous cell lines (BEAS2B, HaCaT, HGF, HEK293T) and tumor cell lines (MDA-MB231, MCF-7, MDA-MB468, SKBR3, BoM1833, HepG2, JHH1, SNU449) were examined by real-time PCR analysis. *n* = 4–10 **p *<* 0.01 compared to the expression in HaCaT cells. **c** The Yap protein levels in immortalized non-cancerous cell lines (BEAS2B, HaCaT, HGF, HEK293T) and tumor cell lines (MDA-MB231, MCF-7, MDA-MB468, SKBR3, BoM1833, HepG2, JHH1, SNU449) were examined by western immunoblotting. The density of bands were quantified and analyzed with Quantity One program (Bio-Rad). *n* = 4. **p < 0.01 compared to the Yap protein expression of HGF cells. **d** Upper, expression of circYap after the cells transiently transfected with circYap plasmid or vector in B16 mouse melanoma cells, MDA-MB231 human breast cancer cells and HepG2 human liver cancer cells. Lower, expression of circYap was examined with the junction primers (forward primer spanned the back-splicing junction) or non-junction primers (forward and reverse primers were located at the two side of the junction). The levels of circYap were compared to the levels of Yap mRNA and housekeeping gene U6 in HEK293 cells and MDA-MB231 cells. **e** Structure of circYap. The existence of circYap was validated by Sanger sequencing. Red letters and square represent “head to tail” junction of circYap. **f** The expression of circYap (left) and Yap mRNA (right) in MDA-MB231 cells that stably overexpressing circYap was compared to those in vector control cells. An equal amount of RNA was also incubated with or without RNase R for 15 min at 37 °C. The spike-in RNA was added after treatment to serve as internal control. *n* = 6. **p < 0.01 compared to vector control, ^##^*p* < 0.01 compared to mock treatment. **g** Vector or plasmid containing circYap or its linear precursor were transient transfected to HEK293T cells. The junction primers and non-junction primers were used to amplify circYap in wide-type (wt), vector, circYap overexpressed and its linear precursor overexpressed cells. The lower panel of bar graph shows the circYap expression detected by qPCR. *n* = 3 **p < 0.01 compared to the vector control. **h** The vector control and circYap overexpressed cells were cultured in serum-free medium for 3 days and re-culture the cells in 10% FBS medium for 24, 32, 40, 48, 56, 64 and 72 h before sample collection. Yap protein expression was examined by western immunoblotting (left). The density of bands was quantified with Quantity One program (left). *n* = 3 *p < 0.05, **p < 0.01 compared to the Yap protein expression of the corresponding vector control at different time points

We determined circYap levels in the tumors of human breast carcinoma and found that circYap levels were significantly lower in tumor tissues compared to adjacent breast tissues (Fig. 1a). We also examined the levels of circYap relative to several known cirRNAs and found that its level was lower than high abundant cirRNAs circHIPK3 and circCDYL [25], but higher than the recently reported tumor suppressor circCcnb1 [26] (Fig. S1a). We examined circYap levels in breast cancer cells, liver cancer cells and immortalized non-cancer cells and found that circYap levels were lower in cancer cells compared to immortalized non-cancer cells (Fig. 1b). However, Yap protein levels were higher in the invasive cancer cells relative to the non-cancer cells (Fig. 1c). These results implicated the association of circYap and Yap proteins.

To test this, we constructed a plasmid with an insert of circYap (Fig. S1c) and transfected it into cells to generate circYap-overexpressing cells. As expected, a significant elevation of circYap expression was observed in MDA-MB231 cells and HepG2 cells after transient transfection (Fig. 1d). In addition, circYap expression could reach to the similar levels to Yap mRNA and housekeeping gene U6 when circYap was transient transfected in MDA-MB231 cells and to even higher levels in high transfection efficiency cells, such as 293T cells **(**Fig. S1d**)**. To verify correct splicing and circularization, human circYap expressed in murine B16 cells was amplified by RT-PCR with divergent primers and subjected to Sanger sequencing. By using murine cells here, we excluded the interference of endogenous human circYap for Sanger sequencing. The predicted “head-to-tail” junction sequence was confirmed (Fig. 1e), suggesting a successful circularization of exogenous human circYap construct in cells. RNase-R that is resistant by circRNA was added into the total RNA to deplete the linear mRNAs according to previous reports [27, 28] and we found that circYap expressed in MDA-MB231 (Fig. 1f) and HepG2 (Fig. S1e) was resistant to RNase-R digestion. As a control, the down-stream intron signal was mutated, producing a linear Yap construct (linYap), which abolished the circularization activity of the construct (Fig. 1g, construct sequence in Fig. S1c). The product of linYap was sensitive to RNase R treatment (Fig. S1f).

Thereafter, cells were synchronized to allow a similar process of protein synthesis by starvation with serum-free medium for 24 h followed by re-incubation in full media for 24–72 h. Yap protein levels were examined by Western blotting. Significant downregulation of Yap protein was found in each time point (Fig. 1h).

### The circYap inhibited Yap translation but not translocation

**Fig. 2:**
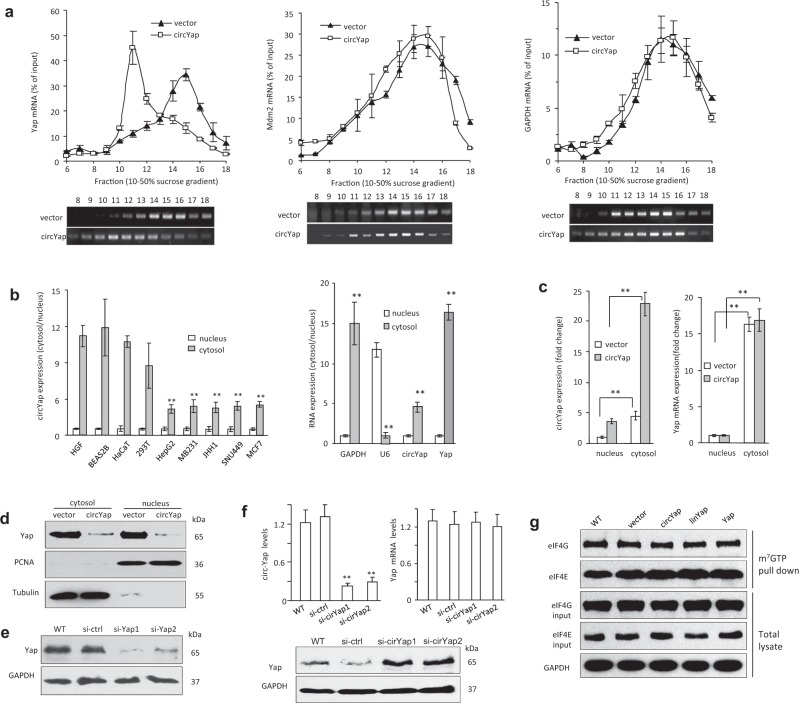
CircYap inhibited the translation of Yap protein. **a** Polysome of the vector control and circYap overexpressed cells were extracted and subjected to 10 to 50% sucrose gradient by ultracentrifuge. Twenty-four polysome fractions were collected from top to bottom followed by RNA extraction. Yap (right), mdm2 (middle) and GAPDH (left) mRNA expression in each fraction were determined by real-time PCR (upper) and visualized by DNA agarose gel (lower). *n* = 3. **b** Left, the cytosolic and nuclear expression of circYap in immortalized non-cancerous cell and tumor cell lines were examined by real-time PCR. The relative ratio of cytosolic to nuclear expression was calculated in different cells respectively. *n* = 6. **p < 0.01 compared to the cytosolic/nuclear ration of circYap in HGF cells. Right, the cytosolic and nuclear expression of circYap and Yap mRNA in HepG2 cells were determined. The expression of cytosolic and nuclear GAPDH and U6 were used to examine the purity of cytosolic and nuclear RNA. *n* = 6. **p < 0.01 compared to nuclear expression of each RNA. **c** The cytosolic and nuclear expression of circYap (left) and Yap mRNA (right) in vector control and circYap overexpressed cells were determined. n = 6. **p < 0.01 compared to vector control. (**d**) The cytosolic and nuclear levels of Yap protein in vector- and circYap-transfected MDA-MB231cells were examined by Western blotting. Tubulin and PCNA antibodies were used to examine the purity of cytosolic and nuclear fractions, respectively. **e** Full length linear Yap mRNA was knocked down by Yap siRNA (si-Yap1 and si-Yap2) in MDA-MB231 cells. Yap protein expression was examined by Western blotting in wide type (wt), siRNA control (si-ctrl), si-Yap1 or si-Yap2. GAPDH protein was examined as loading control. **f** Upper, circYap was knockdown by circYap siRNAs (si-cirYap1 and si-cirYap2) in MDA-MB231 cells. The expression of circYap and Yap mRNA were examined. *n* = 6. **p < 0.01 compared to si-ctrl. Lower, Yap protein levels were examined by Western blotting. **g** General translation rates were examined by cap pull-down assay in wide type (wt), vector, circYap or its linear precursor (linYap), or full-length Yap mRNA overexpressed (Yap) cells. The m7GTP bound eIF4G and eIF4E were detected along with their input in total cell lysate

To investigate whether circYap regulated Yap translation, we conducted a sucrose gradient fractionation assay followed by a RT-qPCR to examine the translation efficiency of Yap upon circYap overexpression. Polysome fraction was isolated followed by RT-qPCR analysis of Yap mRNA expression in each fraction. The Yap mRNA distribution in circYap overexpressed cells shifted from heavy toward light polysomes compared to vector control cells, which indicated a reduction of translation of Yap mRNA (Fig. 2a, left). The distribution of an unrelated mRNA Mdm2 and the housekeeping GAPDH mRNA did not show this pattern (Fig. 2a, middle and right, respectively). As well, transfection of circCcnb1 did not show the shift (Fig. S2a), suggesting specificity of circYap.

To examine whether circYap influenced Yap translocation, cytosolic and nuclear RNA were isolated. Our results showed that circYap was mainly accumulated in the cytosol in both cancer and non-cancer cells (Fig. 2b), while GAPDH was mainly distributed in cytosol (Fig. S2b). After transfecting with circYap construct, both the cytosolic and nuclear circYap expression were elevated (Fig. 2c). In accordance, both the cytosolic and nuclear Yap protein levels were decreased (Fig. 2d, Fig. S2c). These results demonstrated that overexpression of circYap did not drive the cytosol-nuclear translocation of Yap protein. The decrease in Yap expression was similar to silencing endogenous Yap by siRNA approach (Fig. 2e, Fig. S2d).

The circYap siRNAs did not affect expression of Yap mRNA (Fig. 2f, upper), but effectively increased Yap protein levels (Fig. 2f, lower, Fig. S2e). We concluded that circYap negatively regulates Yap protein expression. This appeared to be Yap specific, since the Yap downstream signals CTGF, c-myc and Ccnb1 were down regulated upon circYap expression, but an unrelated gene Mdm2 was not affected (Fig. S2f). Some stress conditions including hydrogen peroxide, doxorubicin, c2-ceramide, and serum deprivation were found to modulate circYap expression (Fig. S2g). We examined general translation rates by polysome profiling and using cap pull-down assay in MDA-MB231 cells transfected with or without vector, circYap, linYap, or full-length Yap mRNA. The m7GTP bound eIF4G and eIF4E were detected along with their input in total cell lysate (Fig. 2g) and polysome profile was analyzed by tracing the absorbance of polysome fraction at 254 nm (Fig. S2h), suggesting expression of circYap had no effect on general translation.

###  circYap suppressed translation initiation of Yap by binding with PABP and eIF4G

**Fig. 3:**
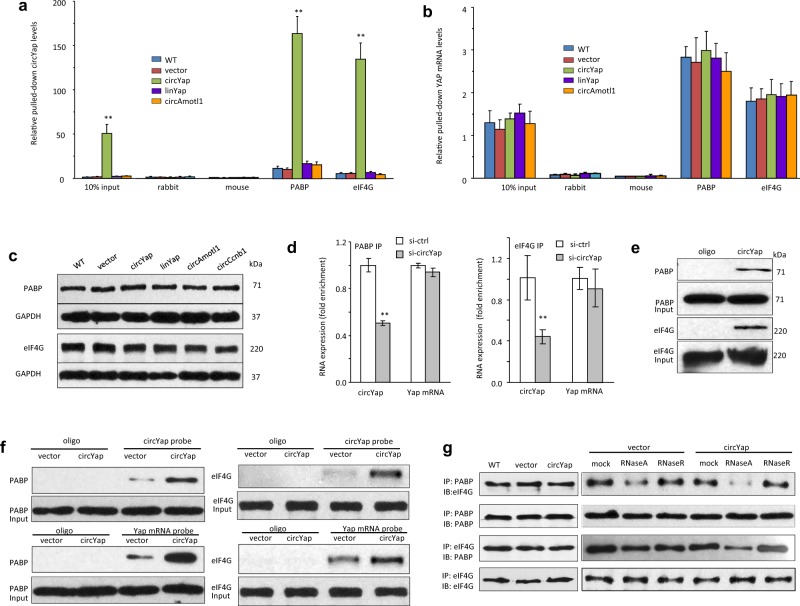
Binding of circYap with PABP and eIF4G protein. **a**, **b** The cell lysate from MDA-MB231 wide-type cells, the cells stably transfected with vector, circYap or its linear precursor (linear Yap), or angiomotin like-1 circRNA (circAmotl1) were incubated with antibody against rabbit or mouse IgG, PABP or eIF4G, and protein A magnetic beads to precipitate RNAs followed by real-time PCR with primers specific for circYap (**a**) or linear Yap mRNA (**b**). *n* = 6. **p < 0.01 compared to corresponding vector control. **c** The expression of PABP and eIF4G were examined in the lysates from MDA-MB231 cells stably transfected with or without vector, circYap, linYap, circAmotl1, or Ccnb1 circRNA (circCcnb1). **d** Lysates from MDA-MB231 cells transfected with si-ctrl or si-circYap were incubated with antibodies against PABP antibodies (left) or eIF4G (right) followed by real-time PCR. *n* = 6. **p < 0.01 compared to siRNA control. **e** MDA-MB231 cell lysates were incubated with biotinylated circYap probe or scramble oligo, and streptavidin beads. The PABP and eIF4G proteins that were pulled down by circYap probe were analyzed by western blotting. *n* = 3. **f** Lysates from MDA-MB231 cells transfected with vector or circYap were incubated with circYap probe or Yap mRNA probe. The pulled down proteins were probed by antibodies against PABP (left) and eIF4G (right). *n* = 3. **g** Left, protein extract from MDA-MB231 cells stably transfected with or without vector and circYap were used for immunoprecipitation (IP) followed by immunoblotting (IB) analysis. Right, after immunoprecipitation (IP) with either PABP or eIF4G antibody, 1/3 of the protein-bound magnetic beads from each group were treated with RNase R to digest linear RNAs and another 1/3 were treated with RNase A to digest both linear and circular RNAs. Then, the beads were washed and the precipitated proteins were eluted with Laemmli buffer followed by western immunoblotting (IB). *n* = 3

Next, we deciphered the possible mechanism by which Yap translation was repressed by circYap. The potential interaction of circYap with translation associated proteins was analyzed and predicated by using different prediction tools, including RPIseq and lncPro (Table S1). The bioinformatic analysis suggested that circYap had promising potential in binding with PABP and eIF4G, two essential members of the translation initiation machinery. We conducted the RNA immunoprecipitation and RNA pull-down assays and found that PABP and eIF4G antibodies could precipitate circYap, but not its linear precursor linYap (Fig. 3a). However, transfection of circYap did not affect binding of PABP and eIF4G to Yap mRNA (Fig. 3b) nor expression of PABP and eIF4G (Fig. 3c). Silencing circYap expression by siRNA decreased the binding ability of circYap with PABP and eIF4G (Fig. 3d). PABP and eIF4G could be pulled-down by the circYap probe (Fig. 3e, Fig. S3a). Overexpression of circYap resulted in pulling-down more PABP and eIF4G by the probe (Fig. 3f, Fig . S3b.). The other components of eIF4F complex including eIF4E, eIF4A and eIF4B could be pulled-down by the circYap probe (Fig. S3c). However, overexpression of circYap did not affect Yap upstream regulators (Mts-1, Mts-2, Lats) (Fig. S3d), nor the other components of eIF4F complex (Fig. S3e) in MDA-MB231 cells.

We conducted immunoprecipitation and immunoblotting (IP-IB) assays with PABP and eIF4G antibodies followed by RNase-A or RNase-R treatment. While transfection of circYap did not affect the interaction of PABP to eIF4G, it significantly reduced the binding of PABP to eIF4G upon treatment with RNase-A in low endogenous circYap-expressing cells MDA-MB231 (Fig. 3g). In addition, when only linear Yap mRNA was degraded by RNase-R, these two proteins could still precipitate each other (Fig. 3g). It suggested that the interaction of PABP with eIF4G during translation of Yap was indirect and bridged by circYap.

### Yap mRNA guided circYap to recognize Yap translation initiation machinery

**Fig. 4:**
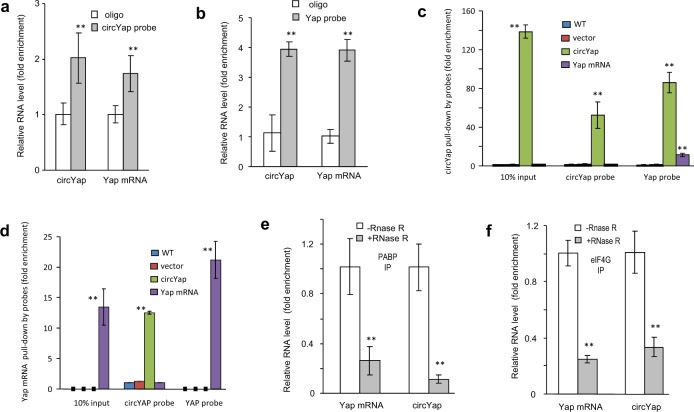
Binding of circYap with linear Yap mRNA. The cell lysates of wide-type MDA-MB231 were incubated with biotinylated scramble oligo, circYap probe (**a**) or Yap mRNA probe (**b**) and streptavidin beads. The circYap or Yap mRNA that were pulled down by the probes were examined by real-time PCR. *n* = 6. **p < 0.01 compared to scramble oligo. **c**, **d** Lysates from MDA-MB231 cells transfected with or without vector, circYap or linear Yap mRNA was incubated with circYap or Yap mRNA probe and streptavidin beads. The circYap (**c**) or Yap mRNA (**d**) that was pulled down by the probes was examined by real-time PCR. *n* = 4. **p < 0.01 compared to vector control. **e**–**f** Lysates from MDA-MB231 cells were treated with or without RNase R for 15 min at 37 °C followed by incubation with an antibodies against PABP (**e**) or eIF4G (**f**) and protein A magnetic beads to precipitate RNAs. The protein-bound RNAs were eluted and the precipitated circYap and Yap mRNA was examined by real-time PCR. *n* = 6. **p < 0.01 compared to mock (-RNase R)

To clarify the reasons behind circYap specifically inhibiting protein translation initiation, we first conducted RNA pull-down assay to examine whether circYap could bind with Yap mRNA and found that Yap mRNA could be pulled-down by the circYap probe (Fig. 4a) and, in turn, circYap could be pulled-down by a probe specifically targeting Yap mRNA (Fig. 4b). In addition, the probes of either circYap or Yap mRNA could pull-down much more of each other in circYap overexpressing cells (Fig. 4c, d). However, overexpression of Yap mRNA could hardly further pull down more circYap because of the much lower levels of circYap compared to Yap mRNA in tumor cells.

To investigate whether binding with Yap mRNA affected the interaction of circYap with PABP and eIF4G, we performed RNase-R treatment to deplete Yap mRNA prior to RNA IP with PABP and eIF4G and detected decreased binding of Yap mRNA with PABP and eIF4G (Fig. 4e, f). The binding of circYap with PABP and eIF4G was also blocked after the RNase-R treatment (Fig. 4e, f), although RNase-R could not break down circRNA. These results suggested that Yap mRNA could bind with circYap and hence, guide the specific anchor of circYap to Yap translation initiation machinery.

### Identification of the binding sites of circYap and Yap mRNA

**Fig. 5:**
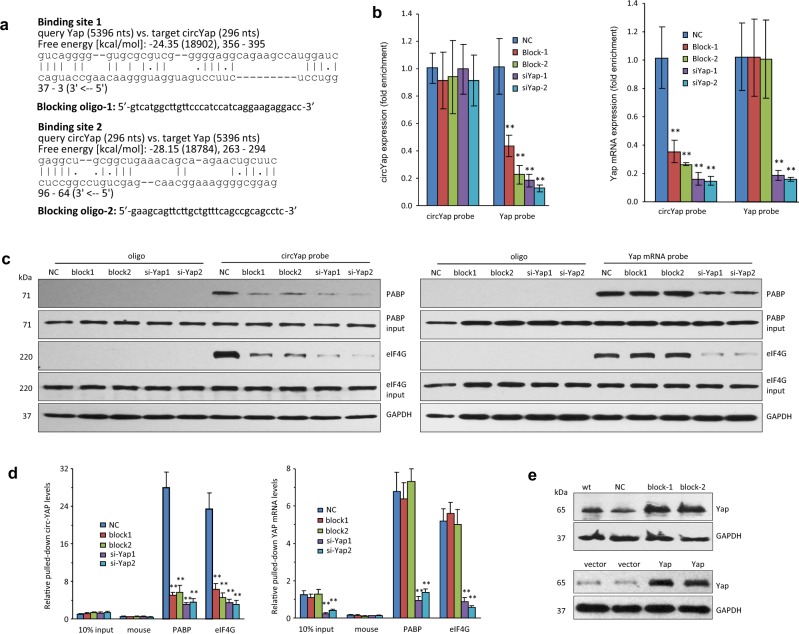
Identification of the binding sites of circYap with Yap mRNA. **a** The binding sites of circYap with Yap mRNA were identified by using the RISearch software. The 2′-O-methyl blocking oligos with complementary sequences were designed. Blocking oligos for the two binding sites (Block-1 and Block-2) or the siRNA for Yap mRNA (siYap-1 and siYap-2) were transfected to MDA-MB231 cells which were then collected 48 h after transfection. The cell lysates from negative control (NC), blocking oligo, or siYap groups were incubated with biotinylated circYap probe or Yap mRNA probe and streptavidin beads. The RNAs were eluted by Trizol reagent from the beads for detecting Yap mRNA and circYap pulled down by probes. The proteins were eluted by RNase-free water containing 0.1% SDS to examining the PABP and eIF4G protein pulled down by probes. **b** The circYap (left) and Yap mRNA (right) levels after pull down by the probes were examined by real-time PCR. *n* = 4. **p < 0.01 compared to negative control (NC). **c** PABP or eIF4G that were pulled down by circYap probes (left) or Yap probes (right) were examined by western blotting. **d** Lysates prepared from cells transfected with negative control (NC), blocking oligo, or siYap were incubated with PABP or eIF4G antibodies and protein A magnetic beads to examine the precipitated circYap (left) and Yap mRNA (right) by real-time PCR. *n* = 4. **p < 0.01 compared to negative control. **e** Yap expression in MDA-MB231 cells transfected with NC and blocking oligos, or transfected with vector or Yap plasmid were examined by western blotting

To further confirm the direct binding of circYap and Yap mRNA, we conducted RIsearch [29] and RNAplex [30] to predict potential binding sites. The results from RIsearch displayed two high affinity binding sites between circYap and Yap mRNA (Fig. 5a), which were located close to the cap of Yap mRNA and the circular junction of circYap. Similar results were confirmed by RNAplex (Fig. S4a). Accordingly, we designed two blocking oligonucleotides targeting the binding site 1 and 2 respectively (Fig. 5a). We transfected the cells with these two blocking oligos followed by RNA pull-down assay with circYap and Yap mRNA probes respectively and found a significant decrease in binding abilities between circYap and Yap mRNA (Fig. 5b). Transfection with these blocking oligos did not affect general protein translation (Fig. S4b), but increased expression of the Yap target genes CTGF, c-myc and Ccnb1 (Fig. S4c). In addition, the blocking oligos decreased the binding of circYap with PABP and eIF4G but could not affect Yap mRNA binding these two proteins (Fig. 5c, Fig. S4d). Transfection with these two blocking oligos also decreased antibodies against PABP and eIF4G precipitating circYap but not Yap mRNA (Fig. 5d). The blocking oligos showed similar effect on reducing the interaction of PABP and eIF4G with circYap compared to siRNAs targeting Yap. Moreover, we demonstrated that blocking the binding sites could functionally elevate expression of Yap to the levels comparable with Yap transfection (Fig. 5e, Fig. S4e).

### Identification of the binding sites of circYap with PABP and eIF4G

**Fig. 6:**
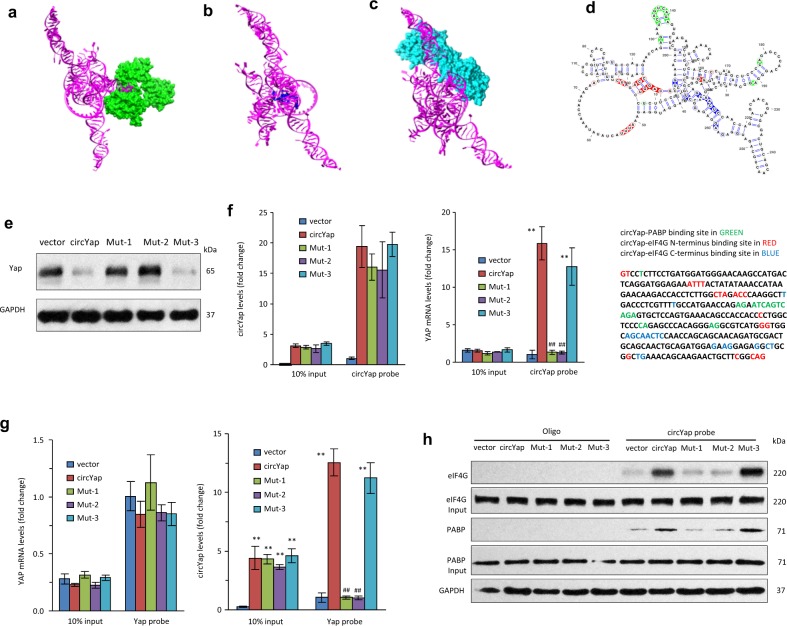
Identify the binding sites of circYap with PABP and eIF4G. The binding sites of circYap with PABP and eIF4G were predicted by NPDock. The interaction sites of protein–nucleic acid structures were calculated by HBPLUS and shown schematically in a diagram generated by the NUCPLOT. Docking model of circYap with PABP and eIF4G was shown as **a** circYap and PABP, **b** circYap and eIF4G (N-terminus), **c** circYap and eIF4G (C-terminus). RNA shows as pink and protein as green (PABP), indigo (eIF4G N-terminus), or blue (eIF4G C-terminus). **d** The secondary structure representation of circ-Yap highlighting the interactive sites with PABP (green), eIF4G (C-terminus) (blue) and eIF4G (N-terminus) (red). **e** The mutation was placed on the binding sites of circYap with Yap mRNA (Mut-1) or two proteins (Mut-2). Yap expression in cells transfected with plasmid containing vector, circYap, mutant binding sites (Mut-1 and Mut-2), or mutant non-essential region (Mut-3) were examined by western blotting. **f** Lysates from MDA-MB231 cells transfected with vector, circYap, Mut-1, Mut-2, or Mut-3 were incubated with circYap probe. The circYap (left) or Yap mRNA (right) pulled down by the probe was examined by real-time PCR. *n* = 4. **p < 0.01 compared to vector control. ^##^*p* < 0.01 compared to circYap overexpressed cells. **g** Lysates from MDA-MB231 cells transfected with vector, circYap, Mut-1, Mut-2, or Mut-3 were incubated with Yap mRNA probe. Yap mRNA (left) or circYap (right) pulled down by the probe was examined by real-time PCR. *n* = 4. **p < 0.01 compared to vector control. ^##^*p* < 0.01 compared to circYap overexpressed cells. **h** Lysates from MDA-MB231 cells transfected with vector, circYap, Mut-1, Mut-2, or Mut-3 were incubated with circYap probe. The pulled down PABP or eIF4G was examined by western blotting

To identify the binding sites of circYap with PABP and eIF4G, we first built the docking model of circYap with PABP and eIF4G. The 3D model of eIF4G (C-terminus) covering 21% of its sequence (1236–1592) was modeled with 100% confidence by the single highest scoring template, and it covered the M2 and W2 domain while the crystal structure of the PABP-binding site (N-terminus) with eIF4G was only part of N-terminal sequence (178–203) [31]. A total of 346 residues (54% of the sequence) of PABP1 were modeled with 100% confidence. A computational docking approach was used to explore how circYap interacts with PABP and eIF4G. The molecular docking models of circYap with PABP (Fig. 6a), eIF4G N-terminus (Fig. 6b) and eIF4G C-terminus (Fig. 6c) were built. The hydrogen bonds and non-bonded interactions between circYap and PABP or eIF4G were shown in Fig. 6d. The different parts of potential bases in circYap interacted with PABP and eIF4G (Fig. 6d). This indicated that circYap could bind both PABP and eIF4G simultaneously. When comparing the binding sites between PABP and eIF4G to the binding sites of circYap on PABP, two overlapping residues were found. Among these binding sites between circYap and PABP, ASN, ASP, PHE, VAL, and MET were located at RRM2 (99–175aa, eukaryotic RNA recognition motif, RRM), which also interacted with PABP and eIF4G. The binding sites of circYap and eIF4G were overlapped with eIF4G-PABP binding sites. These results implied that circYap may block the binding of PABP and eIF4G by competitive inhibition.

A plasmid containing the circYap-Yap mRNA binding site mutation (Mut-1) and a plasmid with the circYap-PABP and circYap-eIF4G binding site mutation (Mut-2) in the circYap were constructed and transfected into cells. An unrelated mutation (Mut-3) was generated. Sequences of mutations are provided in Supplementary (Fig. S5a). Inhibition of Yap expression by circYap was abolished by mutating the binding sites of circYap with Yap mRNA (Mut-1 and Mut2, Fig. 6e, Fig. S5b). Expression of circYap was not affected by these mutations (Fig. S5c). Mutation of these binding sites could block the circYap probe from pulling down Yap mRNA (Fig. 6f) and Yap mRNA probe to pull-down circYap (Fig. 6g). Transfection with Mut-1 and Mut-2 also blocked circYap probe to pull-down PABP and eIF4G (Fig. 6h, Fig. S5d). The inhibition of the 5′- and 3′-UTR translational activity in Yap mRNA by circYap were examined in an in vitro translation system (Fig. S5e-f), which suggested the translational silencing activity of circYap on its parental mRNA.

### The malignant phenotypes of cancer cells were reversed by circYap

**Fig. 7:**
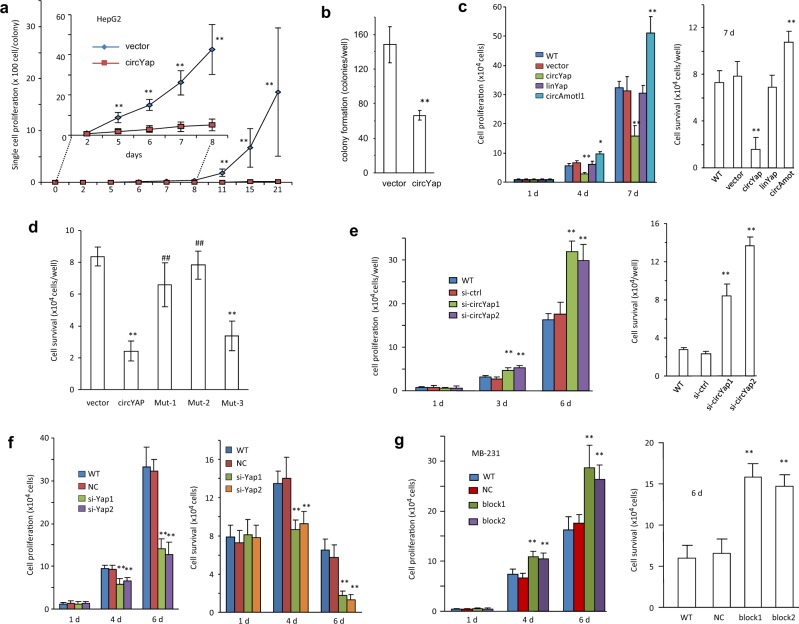
Role of circYap in tumor cell colony formation, proliferation, and survival. **a** HepG2 cells were inoculated in 96-well plates to obtain one cell per well. Single cell proliferation was monitored for up to 21 days. n = 50. **p < 0.01 compared to vector control. Days 0–8 are placed as the inset to show the clear difference between vector and circYap. **b** The HepG2 cells transfected with circYap formed significantly less and smaller colonies than the vector transfected cells. *n* = 6. **p < 0.01. **c** Cell proliferation (left) and cell survival (right) were measured in MDA-MB231 cells stably transfected with or without vector, circYap, linYap, or circAmotl1. *n* = 6. **p < 0.01 compared to vector control. **d** Cell survival were examined in MDA-MB231 cells transfected with vector, circYap, Mut-1, Mut-2 or Mut-3. *n* = 6. **p < 0.01 compared to vector control. ^##^*p* < 0.01 compared to circYap overexpressed cells. **e** Cell proliferation (left) and cell survival (right) were measured in MDA-MB231 cells transfected with or without si-ctrl, si-circYap1 and si-circYap2. *n* = 6. **p < 0.01 compared to siRNA control. **f** Cell proliferation (left) and cell survival (right) were measured in MDA-MB231 cells transfected with or without si-ctrl, si-Yap1 and si-Yap2. *n* = 6. **p < 0.01 compared to siRNA control. **g** Cell proliferation (left) and cell survival (right) were measured in MDA-MB231 cells transfected with or without negative control (NC) or blocking oligos (block-1 and block-2). *n* = 6. **p < 0.01 compared to negative control

We inoculated single cells in each well of 96-well plates and measured cell proliferation for up-to 3 weeks. Both the HepG2 cells (Fig. 7a) and MDA-MB-231 cells (Fig. 7c) overexpressing circYap showed a remarkable inhibition of proliferation. We then conducted flat-plate for 2D monolayer culture and soft-agar for 3D colony formation assays. The size and the numbers of colonies were reduced in circYap-transfected cells (Fig. 7b, Fig. S6a). Lacking circularization abolished the effect of growth inhibition and cell viability (Fig. 7c). Mutations of the binding sites for PABP, eIF4G, and Yap mRNA abolished the inhibitory effect (Fig. 7d). Silencing circYap increased cell proliferation and survival (Fig. 7e), while silencing endogenous Yap produced opposite effects (Fig. 7f). Transfection with the blocking oligos promoted cell proliferation and survival (Fig. 7g).

**Fig. 8:**
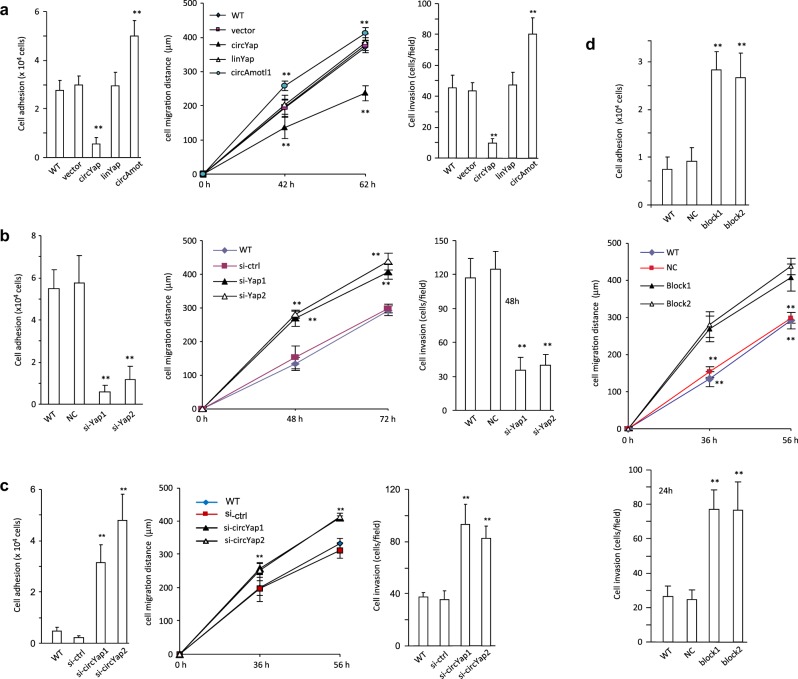
Role of circYap in tumor cell migration, invasion, and adhesion. **a** MDA-MB231 cells were stably transfected with vector, circYap, linYap, or circAmotl1. Left, the cells were inoculated in Petri dishes. Adhesive cells were counted 24 h after inoculation. *n* = 6. **p < 0.01 compared to vector. Middle, cell migration was examined with a scratch migration assay. Five pairs of random points were selected for measuring the migrating distances in each scratch. *n* = 6. **p < 0.01 compared to vector. Right, MDA-MB231 cells were stably transfected with or without vector, linYap or circAmotl1. Cell invasion was determined by loading 1 × 10^5^ cells in trans-wells with 10% Matrigel in serum-free medium followed by incubation in the 24-well plates with 10%FBS medium during 48 h. *n* = 6. **p < 0.01 compared to vector. **b** MDA-MB231 cells were transfected with or without si-ctrl, si-Yap1 or si-Yap2, followed by cell adhesion (left), migration (middle) and invasion (right) assays. *n* = 6. **p < 0.01 compared to siRNA control. **c** MDA-MB231 cells were transfected with or without si-ctrl, si-circYap1 or si-circYap2, followed by cell adhesion (left), migration (middle) and invasion (right) assays. *n* = 6. **p < 0.01 compared to siRNA control. **d** MDA-MB231 cells were transfected with or without NC, block-1 or block-2, followed by cell adhesion (left), migration (middle) and invasion (right) (right) assays. *n* = 6. **p < 0.01 compared to negative control

**Fig. 9:**
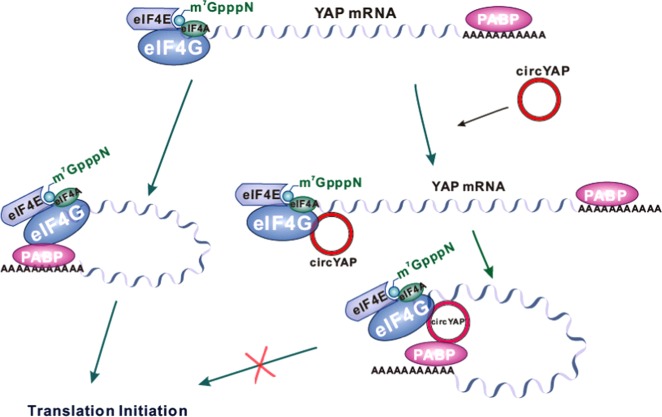
Model depicting the proposed mechanism of suppression of translation initiation by circYap. In the translation initiation complex, eIF4G binds with PABP leading to mRNA circularization and bolster translation. The circYap could specifically recognize and bind with Yap mRNA, and meanwhile bind with eIF4G and PABP which are responsible for Yap translation initiation. Such bindings competitively inhibit the interaction of eIF4G and PABP and therefore suppress the translation initiation of Yap

In addition, ectopic circYap decreased cell adhesion, migration, and invasion (Fig. 8a, Fig. S6b, Fig. S7a), similar to silencing endogenous Yap (Fig. 8b, Fig. S7b). Silencing endogenous circYap produced opposite effects on cell adhesion, migration and invasion (Fig. 8c, Fig. S6c, Fig. S7c). Transfection with the blocking oligos increased cell adhesion, migration, and invasion (Fig. 8d, Fig. S7d). These results suggested that unlike linear Yap mRNA, circYap played an opposite role in cancer cell activities. Taken together, we reported for the first time that circRNA could specifically and directly bind with its parental mRNA, silencing the translation of its own protein and thus regulating its parental gene’s activities (Fig. 9).

## Discussion

Here, we present evidence that Yap translation is negatively controlled by its circRNA via suppressing the formation of translational initiation machinery. One of our main findings is that circYap directly suppresses the assembly of Yap translation initiation machinery, leading to the suppression of Yap translation, and thus decelerates tumor cell progression. It is well-established that Yap plays a critical role in tumorigenesis [32, 33]. Exploring selective and direct inhibitory mechanisms of Yap function may have better therapeutic efficacy with minimal side effects. In light of the available data, we postulate that suppression of translation initiation by circYap is a novel mechanism for silencing Yap directly and selectively. Consistent with the findings from previous studies in breast, liver, and lung cancers, our results showed that Yap protein was highly expressed in breast and liver cancer cells compared to immortalized non-cancer cells. Meanwhile, circYap expression was significantly suppressed in these cancer cells. It suggested that the abnormal high expression of Yap in cancers might be resulted from the deregulation of circRNA expression since we found a significant reduction of circYap in tumor cells. Our results supported that circYap could be a promising tool for antagonizing Yap during cancer therapy.

We deciphered the possible molecular mechanism by focusing on circYap and its ability to regulate the translation of Yap. Dysregulation of translation is considered as a hallmark of cancer and is associated with aberrant proliferation, survival, apoptosis, angiogenesis, and cancer energetics [11, 34]. By taking a computational approach, we reached the conclusion that eIF4G and PABP were ranked highly compared to other translation related proteins. The binding of circYap with eIF4G and PABP was further confirmed. The importance of the eIF4G-PABP interaction for efficient translation initiation has been well established in many systems [35]. In the complex, eIF4G binds PABP, leading to mRNA circularization which  bolsters translation [36]. Overexpression of eIF4G has been reported to correlate with breast cancer malignancies [37]. Previous studies discovered that PABP-interacting protein 2 (Paip2) competed with eIF4G for binding to PABP with a shared sequence of PABP (RRM2) [38]. Herein, we reported on the existence of an alternative mechanism by which circYap inhibits the translation via competing with eIF4G for binding to PABP. Our results showed that circYap was able to bind to linear mRNA and these two translational initiation factors. Notably, in the translational initiation complex containing exogenous circYap, the binding of PABP on the poly(A) tail and eIF4G on the 5′-cap of the mRNA was blocked, which may have adversely affected the circularization of Yap mRNA and eventually stalled the translational initiation.

Nonetheless, eIF-PABP complex generally controls the translation initiation of all mRNAs. We asked whether circYap specifically abrogated Yap mRNA translation. Our results implied that circYap would recognize Yap mRNA and inhibit Yap translation. Next, we tried to identify the specific binding sites and tested whether manipulating these binding sites would abolish the effects of circYap on the assembly of translational initiation machinery. Using bioinformatic methods, we found two potential binding sites of circYap and Yap mRNA with the free energy as low as −24 to −28 kcal/mol, which suggested a tight interaction of circYap and Yap mRNA. We also identified the potential binding sites of circYap with PABP or eIF4G. By using blocking oligonucleotides or mutant construct targeting these binding sites, we blocked the interplay between circYap and Yap mRNA or the binding of circYap with translational initiation factors. Thereafter, the binding between circYap and translation machinery was abolished, leading to an elevation of Yap translation followed by an escalation of cancer cell survival and migration. In addition, we noticed mutation of the PABP and eIF4G binding sites on circYap also significantly reduced the binding ability of circYap and Yap mRNA. We assumed such reduction might be resulted from the following two reasons. Firstly, the binding sites of circYap with Yap mRNA were partially overlapped with its binding sites with PABP and eIF4G (Fig. S5a). Therefore, mutations of these binding sites would not only block the binding of circYap with PABP and eIF4G but also disturb the binding of circYap with Yap mRNA, if these overlapping sites played essential roles in maintaining the spatial structure of circYap and its affinity with Yap mRNA. Secondly, the reduction may be due to blocking the binding of circYap with the PABP and eIF4G via nucleotide mutation, resulting in increased affinity and stability of translation machinery and therefore compromised the affinity of circYap and Yap mRNA. These results validated that circYap specifically recognized and bound with its parental mRNA, thus obstructing the assembly of translation machinery. Our previous studies have demonstrated circRNA could bind with functional proteins and regulate tumorigenesis [14, 15]. Here, for the first time, we reported that circRNA could specifically and directly bind with its parental mRNA. This action silenced mRNA translation. Blockage of the translation machinery assembly by circRNA is a novel and unique mechanism from microRNA sponge function of circRNA regarding protein synthesis inhibition. Ultimately, this raises a question whether it is unique for circYap to bind its parental linear mRNA. We predict  that the binding is highly dependent on the secondary and three-dimensional structures of circRNA and its parental mRNA. In our previous studies, we reported that overexpression of some circRNAs, such as circAmotl1 and circFoxo3, could not suppress their protein levels [14, 15]. Therefore, further investigation is required to determine whether other circRNAs have this capability.

Taken together, our study uncovered a novel mechanism underlying the direct regulation of Yap protein expression at translational level and provided a molecular basis for the new understanding of the pathophysiological function of circYap. Furthermore, because of the critical role of Yap in tumorigenesis, our findings may also lay a foundation for the pursuit of circYap as a potential tool for cancer intervention.

## Materials and methods

### Cell culture, transfection, small interfering RNAs, and plasmid

Human breast cancer cell line MDA-MB231 and human liver cancer cell line HepG2 were used for transfection and functional tests. Murine melanoma cell line B16 was used for transfection and circular junction sequencing. All cells were cultured in DMEM supplemented with 10% FBS, Penicillin and Streptomycin. All siRNAs, including control siRNA (5′-uucuccgaacgugucacguuu) and two siRNAs to circYap (5′cugcuucggcagguccucuuu and 5′gcuucggcagguccucuucuu) were transfected at 20 nM final concentration and analyzed 48 h later. The plasmid expressing linear Yap mRNA was purchased from Addgene. The plasmids containing mutant binding sites were generated by Gene Universal.

### Construct generation

A construct expressing human circular RNA Yap1 (circ-Yap1) was generated by us. The plasmids contained a Bluescript backbone, a CMV promoter driving human circYap1 or a non-related sequence serving as a control. The green fluorescent protein (GFP) expression unit was linked to the circYap1 which contained a separate CMV promoter.

### Routine in vitro and in vivo assays

Cell proliferation, survival, migration and adhesion were performed as previously described [39]. In single cell proliferation assay, cells transfected with circYap and control vector were inoculated in Petri dishes in DMEM containing 10% FBS, which allowed the cells to attach but not spread as it was observed in the tissue culture plates. The cultures were briefly treated with trypsin/EDTA in the following day to harvest single cell suspension. The cell number was determined to obtain a density of one cell per 100 µl followed by immediate distribution into 96-well tissue culture plates at the amount of 100 µl per well. The wells that contained single cell were used. Cell number was determined daily. Consent for human samples was obtained according to the Declaration of Helsinki.

### RNase treatment

RNase treatment was conducted as previously described [28]. For spike-in assay, 2 μg total RNA was incubated with 3 U/μg RNase R (Epicentre) for 15 min at 37 °C. Another 2 μg RNA was incubated at the same conditions to be used for mock treatment. Then, the RNA was spiked with 10% mouse RNA and extracted with Phenol/Chloroform followed by ethanol precipitation. The RNA concentrations of the mock-treated samples were determined. One microgram RNA of mock-treated samples and the same volume of the RNase R-treated samples was used for reverse transcription. The cDNA was used for qPCR quantification. The Ct value of GAPDH for mock treatment was used for both RNAse R-treated and mock-treated samples due to degradation of linear RNAs in the RNase R-treated samples. For other assay, 1 μg RNase R (Epicentre) or RNase A (Qiagen) was added in the mixture either before or after immunoprecipitation and incubated for 15 min at 37 °C.

### RT-PCR

Total RNA was extracted from cells using a kit from Qiagen. 1 μg RNA was subjected to reverse transcription and quantitative PCR (qPCR) using iScript RT kits and SYBR green master mix (Bio-Rad). The U6 or GAPDH were used as an internal control. The sequences of primers were listed in the Supplementary Table S2.

### Western blot

Yap protein levels were determined by western immunoblotting analysis. In brief, proteins isolated from cells (40 μg) were separated by electrophoresis on a 10% SDS polyacrylamide gel. Partitioned proteins were transferred to a nitrocellulose membrane. The membrane was probed with rabbit anti-Yap antibody (Cell Signaling Technology), rabbit anti-PABP antibody (Abcam), rabbit anti-eIF4G antibody (Cell Signaling Technology), mouse anti-eIF4A, anti-eIF4B or anti-eIF4E (Santa Cruz Biotechnology). HRP-conjugated anti-rabbit or anti-mouse IgG antibody (Jackson ImmunoResearch) was used as the secondary antibody. The corresponding protein bands were visualized using enhanced chemiluminescence reagents. The same membranes were re-probed with HRP conjugated GAPDH antibody (Proteintech), mouse anti-alpha tubulin antibody (Santa Cruz Biotechnology) or mouse anti-PCNA (Santa Cruz Biotechnology) to confirm equal loading of proteins for each sample.

### Sucrose density gradient assay

Sucrose density gradient assay was used to determine the mRNA translation as described before [40]. In brief, polysome were prepared in 500 μl of hypotonic buffer containing 5 mM Tris-HCl (pH 7.5), 2.5 mM MgCl_2_, 1.5 mM KCl, 1× protease inhibitor cocktail (EDTA-free), 0.5% Triton X-100, 0.1 mg/ml cycloheximide and 0.5% sodium deoxycholate. The polysome lysate were centrifuged at 16,000 × *g* for 7 min at 4 °C and supernatant was collected. The 5, 10, 20, 30, 40, and 50% sucrose solutions were made and filled in ultracentrifuge tube according to the density. The polysome supernatant was loaded carefully on top of the sucrose gradient solution followed by ultracentrifuge at 28,000 rpm for 2 h at 4 °C. Then, the sucrose gradient was collected from top to bottom at 1.5 ml per tube and the UV absorbance was determined at 254 nm. In addition, the total RNA in each tube was isolated and the RNA expression of Yap in each fraction was determined by real-time PCR.

### Cap binding pull down assay

The cap-binding pull down assay was conducted in transfected MDA-MB231 cells as described [41]. In brief, cells were lysed in IP buffer (Tris-HCl pH 7.5 50 mM, NaCl 150 mM, EDTA 1 mM, EGTA 1 mM, TritonX-100 1%, and NP-40 0.5%) containing protease inhibitors (Calbiochem). Total protein extract (1 mg) was incubated with 20 μl m7GpppG conjugated Sepharose beads (AC-155, Jena Bioscience) overnight at 4 °C with gentle rotation. Following pull down, the beads were washed, and the cap bound proteins were eluted by Laemmli buffer. The eIF4E and eIF4G were determined by Western blotting.

### RNA immunoprecipitation

RNA immunoprecipitation was used to determine the binding of RNA and protein. Briefly, cells were lysed in 200 µl co-IP buffer. The total protein lysate was collected and the protein concentrations of different samples were equalized. One tenth of the equalized protein lysates were saved as input for further experiments. The magnetic beads (Surebeads, Bio-Rad) were washed with PBST (PBS containing 0.1% Tween 20) and incubated with 5 μg of primary antibody at room temperature for 10 min. After being washed, the beads were mixed with protein lysis and incubated for another 1 h. Then the beads were washed three times with PBST and resuspended in 0.5 ml Trizol (Invitrogen) for RNA extraction. The eluted co-precipitated RNA or input RNA in the aqueous solution was subject to qRT-PCR analysis to demonstrate the presence of the binding products using respective primers. The co-precipitated circYap or Yap mRNA levels were normalized with the house-keeping gene U6 levels of the corresponding input.

### RNA pull down assay

The pull-down assay was performed using an RNA probe as described [21]. In brief, the cells were lysed in co-IP buffer and then incubated with 3 μg biotinylated DNA oligo probes against circ-Yap or Yap mRNA at room temperature for 2 h. Fifty microliters of Streptavidin C1 magnetic beads (Invitrogen) were added to each binding reaction and further incubated at room temperature for another 1 h. The beads were washed briefly with co-IP buffer for five times. The bound proteins in the pull-down material were analyzed by western blotting. The oligomers for RNA pull-down of human circYap (5′-tcaggaagaggacctgccgaagcagttcttgc) and Yap mRNA (5′-gttcatcatattctgctgcactggtggactgg) were biotinylated at the 5′ end.

### Bioinformatics prediction

The secondary structure of circYap was formed by RNAfold [42]. Based on its circular 2D folding form, the tertiary structure without closed circular could be formed by using RNAComposer method [43]. Crystal structure of the PABP-binding site of eIF4G in complex with RRM1-2 of PABP and poly(A) (PDB ID: 4F02) was downloaded from the Protein Data Bank [44]. Then the complexes of protein–nucleic acid structures were predicted by NPDock. Its computational workflow includes docking, scoring of poses, clustering of the best-scored models and refinement of the most promising solutions [45]. These docking models were clustered according to their mutual similarity with the threshold of 5 Å and the best-scored model from the selected clusters will be chosen for further analysis. The interaction sites of protein-nucleic acid structures were calculated by HBPLUS [46] and shown schematically in a diagram generated by the NUCPLOT [47]. In addition, the interaction sites were shown on the secondary structure of circRNA produced by VARNA program [48].

### In vitro translation

The plasmid encoding luciferase T7luc(A) and Yap mRNA with 5′ and 3′ UTR was constructed by Gene Universal according to previous description [49]. The in vitro transcription and translation were performed based on previous report with minor modification [50]. The plasmid was digested with BamHI and transcribed with T7 RNA polymerase (Thermo Fisher Scientific). Capped RNA transcripts were synthesized in vitro using the MAXIscript T3 kit (Thermo Fisher Scientific) with the presence of m7GTP cap analog (Thermo Fisher Scientific). RNA purification was performed according to manufacturer’s instruction. Rabbit reticulocyte lysate (Promega) was incubated with 0.8 mM elastatinal (Abcam) on ice for 10 min followed by mixing with RNA extracts from cells with or without circYap overexpression. The mixtures were incubated with capped luciferase RNA at 30 °C for 60 min. Luciferase activity of the translation mixture were examined by using Luciferase assay system (Promega) in luminescence counter (Perkin Elmer).

## Supplementary information

circYAP-Supplementary-Table 1

circYAP-Supplementary-Table 2

circYAP-Supplementary-Fig S1

circYAP-Supplementary-Fig S2

circYAP-Supplementary-Fig S3

circYAP-Supplementary-Fig S4

circYAP-Supplementary-Fig S5

circYAP-Supplementary-Fig S6

circYAP-Supplementary-Fig S7
