# Supplementary material for: Translation of yes-associated protein (YAP) was antagonized by its circular RNA via suppressing the assembly of the translation initiation machinery
Source: Cell Death Differ. 2019 May 15;26(12):2758–73. doi: 10.1038/s41418-019-0337-2 (PMC7224378; doi:10.1038/s41418-019-0337-2)
Supplement: Supplementary file 1 — circYAP-Supplementary-Table 1 [file 41418_2019_337_MOESM1_ESM.pdf]

Supplementary Table S1. Prediction of the binding potential of circYAP and translation associated proteins.

CircRNA	Protein	RPISeq		lncPro
		RF	SVM	Value
circYAP1	mTOR	0.50	0.70	71.02
circYAP1	PABPC1	0.60	0.53	90.81
circYAP1	PABPC3	0.60	0.58	88.06
circYAP1	PABPC4	0.55	0.33	90.86
circYAP1	PABPC5	0.60	0.42	75.85
circYAP1	eIF2A	0.55	0.51	82.19
circYAP1	eIF2B1	0.60	0.43	54.84
circYAP1	eIF2B2	0.65	0.48	56.90
circYAP1	eIF2B3	0.60	0.30	68.62
circYAP1	eIF2B4	0.70	0.56	53.75
circYAP1	eIF2B5	0.70	0.31	72.81
circYAP1	eIF2D	0.60	0.61	93.79
circYAP1	eIF2S1	0.55	0.20	90.23
circYAP1	eIF2S2	0.55	0.27	81.47
circYAP1	eIF2S3	0.60	0.27	50.83
circYAP1	eIF4A2	0.55	0.58	70.37
circYAP1	eIF4A3	0.60	0.39	66.31
circYAP1	eIF4B	0.50	0.36	94.14
circYAP1	eIF4E	0.70	0.42	50.47
circYAP1	eIF4G	0.65	0.67	78.94
circYAP1	eIF4G1	0.65	0.65	78.91
circYAP1	eIF4G2	0.70	0.46	73.60
circYAP1	eIF4G3	0.65	0.54	76.36
circYAP1	eIF4H	0.60	0.28	80.59
circYAP1	eEF1A1	0.50	0.24	74.36
circYAP1	eEF1A2	0.60	0.40	76.96
circYAP1	eEF1B2	0.65	0.31	84.57
circYAP1	eEF1D	0.70	0.23	66.53
circYAP1	eEF1E1	0.75	0.33	34.48
circYAP1	eEF1G	0.90	0.64	56.63
circYAP1	eEF2	0.70	0.49	77.13
