# Supplementary material for: Translation of yes-associated protein (YAP) was antagonized by its circular RNA via suppressing the assembly of the translation initiation machinery
Source: Cell Death Differ. 2019 May 15;26(12):2758–73. doi: 10.1038/s41418-019-0337-2 (PMC7224378; doi:10.1038/s41418-019-0337-2)
Supplement: Supplementary file 2 — circYAP-Supplementary-Table 2 [file 41418_2019_337_MOESM2_ESM.pdf]

Supplementary Table S2. Primer sequences.

Primer name		Primer Sequences
circYap junction	Forward:	5’-GCAAGAACTGCTTCGGCAGGTCCT-3’
	Reverse:	5’-GTTTATATAGTAAATTTCTCCATC-3’
circYap non-junction	Forward:	5'-GCAGCAACTGCAGATGGAGAAGGAG-3'
	Reverse:	5'-GGGTCTAGCCAAGAGGTGGTCTTG-3'
circYap linear precusor	Forward:	5'-TCTTCCTGATGGATGGGAAC-3'
	Reverse:	5'-GGCTGTTTCACTGGAGCACT-3'
Yap mRNA	Forward:	5’-CCGTGCCCATGAGGCTCCGGAAGC-3’
	Reverse:	5’-GGGTGTAGCTGCTGGGCCAGAGAC-3’
circHIPK3	Forward:	5'-TATGTTGGTGGATCCTGTTCGGCA-3'
	Reverse:	5'-TGGTGGGTAGACCAAGACTTGTGA-3'
circCDYL	Forward:	5'-CTGTTCCGGCTCCCAAGTGT-3'
	Reverse:	5'-AGGCTTAGCTGTTAACGGG-3'
circFoxo3	Forward:	5'-GTGGGGAACTTCACTGGTGCTAAG-3'
	Reverse:	5'-ATTGTCCATGGAGACAGCCCGCCG-3'
circCcnb1	Forward:	5'-TGATCGGTTCATGCAGGTTGATAC-3'
	Reverse:	5'-TCCAGATGTTTCCATTGGGCTTGG-3'
Mdm2	Forward:	5'-ATGGTGAGGAGCAGGCAAATGTGC-3'
	Reverse:	5'-GCTTCTCATCATATAATCGTTTAG-3'
CTGF	Forward:	5'-CAAGGGCCTCTTCTGTGACT-3'
	Reverse:	5'-ACGTGCACTGGTACTTGCAG-3'
c-myc	Forward:	5'-CGGGTAGTGGAAAACCAGGTAAGC-3'
	Reverse:	5'-TTTCCCTCTGCCTTCTCCTCTCCC-3'
Ccnb1	Forward:	5'-GGTACCTATGCTGGTGCCAGTGCC-3'
	Reverse:	5'-CATCAGAGAAAGCCTGACACAGGT-3'
GAPDH	Forward:	5'-AAAGCTGGGGCTCATTTGCAG-3'
	Reverse:	5'-GATGTTCTGGAGAGCCCCGCG-3'
U6	Forward:	5'-GTGCTCGCTTCGGCAGCACATA-3'
	Reverse:	5'-TGGAACGCTTCACGAATTTGCG-3'
