# Supplementary material for: Translation of yes-associated protein (YAP) was antagonized by its circular RNA via suppressing the assembly of the translation initiation machinery
Source: Cell Death Differ. 2019 May 15;26(12):2758–73. doi: 10.1038/s41418-019-0337-2 (PMC7224378; doi:10.1038/s41418-019-0337-2)
Supplement: Supplementary file 3 — circYAP-Supplementary-Fig S1 [file 41418_2019_337_MOESM3_ESM.pdf]

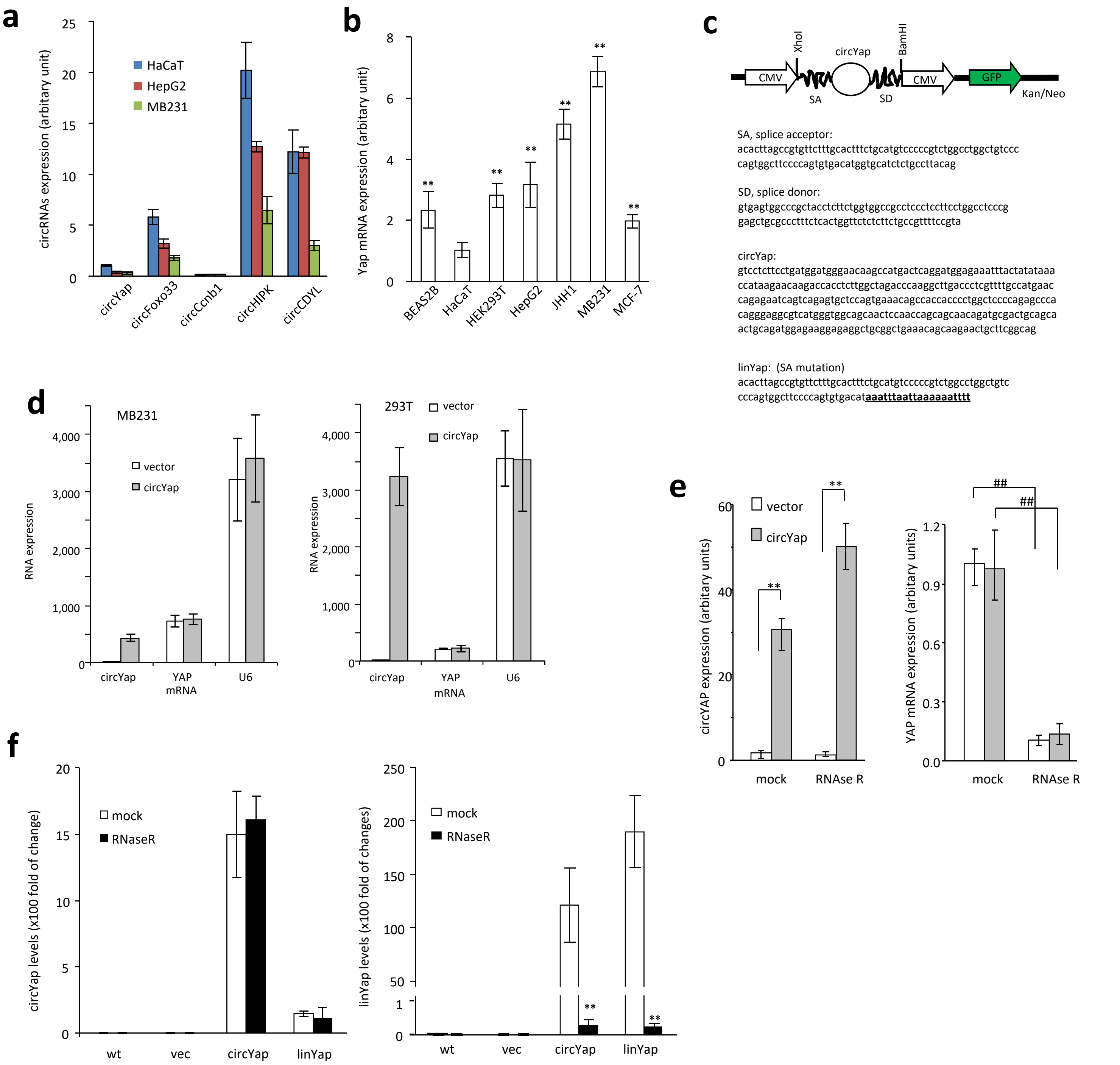

Supplementary Figure S1: Construction of circYAP plasmid.

(a) The relative expression levels of circYAP to other circular RNAs in cancer and non-cancerous cell lines. n=4.

(b) The relative expression of YAP mRNA in cancer and non-cancerous cell lines. n=4. $^{**}p<0.01$ compared to HaCaT cells. Yap mRNA expression in HaCaT cells was significantly lower compared to other immortalized non-cancerous cell lines (BEAS2B and HEK293T) and cancer cell lines (HepG2, JHH1, MDA-MB231 and MCF-7). No significant positive or negative correlation was found between Yap mRNA and circYap. The Yap mRNA levels appeared to be related to high proliferation rate.

(c) Structure and sequence of circYap construct.

(d) The relative expression of circYap, Yap mRNA and U6 in the same sample was examined by Real-time PCR in MDA-MB231 cells (left) and HEK293T cells (right) with transient transfection of vector and circYap plasmid. n=3. The endogenous linear Yap mRNA level was about a thousand times higher than endogenous circYap levels in MDA-MB231 cells. After transient transfection, the circYap expression reached to about 10 times higher compared to endogenous linear Yap mRNA levels in MDA-MB231 cells and HEK293T cells depending on the transfection efficiency of the cells.

(e) The expression of circYAP (left) and YAP mRNA (right) in HepG2 cells after stable transfection with circYAP plasmid. An equal amount of RNAs (2μg) from the cells were incubated with or without RNase R for 15 minutes at 37°C. The spike-in RNA was added after treatment to serve as internal control. n=6. $^{**}p<0.01$ compared to vector control, ## $p<0.01$ compared to mock treatment.

(f) The expression of circYap and its linear precursor in MDA-MB231 cells after transient transfection of circYap plasmid, linear precursor plasmid as well as vector plasmid. Cells were transient transfected with plasmids and collected for RNA extraction after 48h of transfection. An equal amount of RNAs (2μg) from cells were incubated with RNase R or mock treated for 15 minutes at 37°C. The spike-in RNA was added after treatment to serve as internal control. n=6. $^{**}p<0.01$ compared to mock treatment. The levels of circYap were not decreased after RNase R treatment compared to mock treated group, while its linear precursor Yap levels were remarkably reduced upon RNase R treatment. These results suggested circYap are not sensitive to RNase R.
