# Supplementary material for: Translation of yes-associated protein (YAP) was antagonized by its circular RNA via suppressing the assembly of the translation initiation machinery
Source: Cell Death Differ. 2019 May 15;26(12):2758–73. doi: 10.1038/s41418-019-0337-2 (PMC7224378; doi:10.1038/s41418-019-0337-2)
Supplement: Supplementary file 4 — circYAP-Supplementary-Fig S2 [file 41418_2019_337_MOESM4_ESM.pdf]

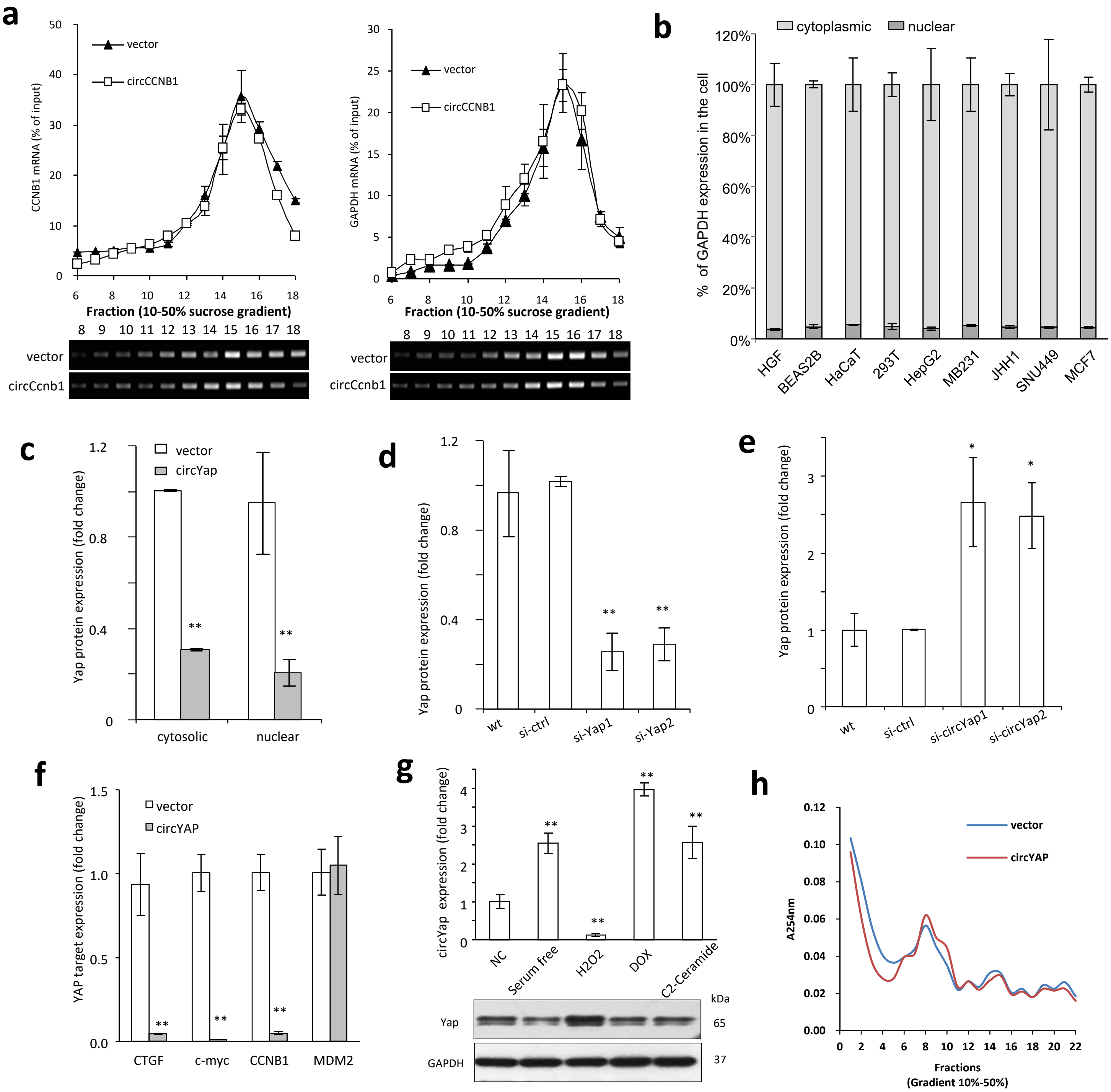

Supplementary Figure S2: The circYap inhibited Yap protein expression.

(a) Polysome of the vector control and circCCNB1 overexpressed cells were extracted and subjected to 10% to 50% sucrose gradient by ultracentrifuge. Twenty-four polysome fractions were collected from top to bottom followed by RNA extraction. CCNB1 (right) and GAPDH (left) mRNA expression in each fraction were determined by real-time PCR (upper) and visualized by DNA agarose gel (lower). n=3. These results showed that circCCNB1 could not affect CCNB1 protein synthesis, indicating that not all the circRNAs could bind to its mRNA and translation initiation machinery to suppress the translation initiation. The function of circYap in regulating translation initiation is specific for its own protein.

(b) The cytosolic and nuclear expression of GAPDH in non-cancerous cell and tumor cell lines were examined by real-time PCR analysis to determine the purity of the cytosolic and nuclear RNA.

(c) The densitometry of the blots in Figure 2d was analyzed with Quantity One program (Bio-Rad). n=3. **p<0.01 compared to vector control.

(d) The densitometry of the blots in Figure 2e was analyzed with Quantity One program (Bio-Rad). n=3. **p<0.01 compared to siRNA control.

(e) The densitometry of the blots in Figure 2f was analyzed with Quantity One program (Bio-Rad). n=3. **p<0.01 compared to siRNA control.

(f) The expression of Yap protein target genes (CTGF, c-myc and CCNB1) and non-Yap target gene (Mdm2) was examined by real-time PCR in MDA-MB231 stably transfected with vector or circYap plasmid. n=4. **p<0.01 compared to vector control. These results suggested the role of circYap in suppressing Yap protein synthesis may further affect the transcription of Yap target genes, since Yap protein is the co-transcription factor of CTGF, c-myc and CCNB1.

(g) The expression of circYap under stress condition. The MDA-MB231 cells were treated with 300 µM hydrogen peroxide (H_2O_2), 50 ng/ml doxorubicin (DOX), 2 µM c2-ceramide, or serum deprivation for 2 hours. The circYap expression was detected by real-time PCR and YAP protein expression was detected by Western immunoblotting. n=4. **p<0.01 compared to non-treated negative control (NC).

(h) The Absorbance at 254 nm of these polysome fractions were measured by spectrophotometer. n=2. The polysome profile suggested that circYap did not affect the general translation.
