# Supplementary material for: Translation of yes-associated protein (YAP) was antagonized by its circular RNA via suppressing the assembly of the translation initiation machinery
Source: Cell Death Differ. 2019 May 15;26(12):2758–73. doi: 10.1038/s41418-019-0337-2 (PMC7224378; doi:10.1038/s41418-019-0337-2)
Supplement: Supplementary file 5 — circYAP-Supplementary-Fig S3 [file 41418_2019_337_MOESM5_ESM.pdf]

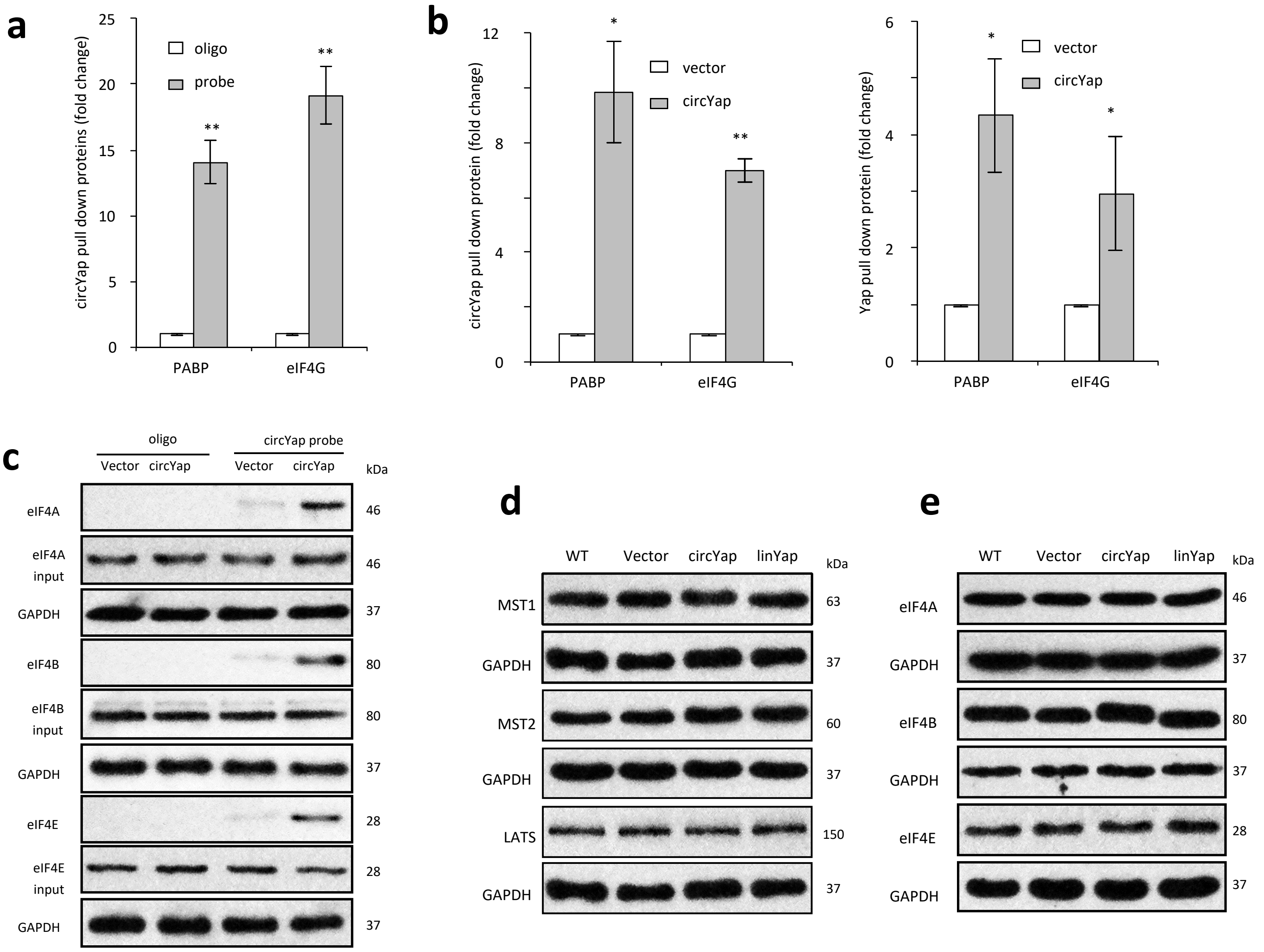

Supplementary Figure S3: Binding of circYap with PABP and eIF4G protein.

(a) The densitometry of the blots in Figure 3e was analyzed with Quantity One program (Bio-Rad). The density of the bands was normalized by PABP or eIF4G input. n=3. **p<0.01 compared to scramble oligo.

(b) The densitometry of the blots in Figure 3f was analyzed with Quantity One program (Bio-Rad). The densities of the bands were normalized by PABP or eIF4G input. The left panel showed the PABP and eIF4G protein pulled down by circYap probe. The right panel showed the PABP and eIF4G protein pulled down by Yap mRNA probe. n=3. **p<0.01 compared to vector control.

(c) MDA-MB231 cell lysates from vector control or circYap-transfected cells was incubated with biotinylated circYap probe or Yap mRNA probe and streptavidin beads. The eIF4A, eIF4B and eIF4E proteins that were pulled down by circYap probe were examined by western immunoblotting.

(d) Protein expression of YAP upstream regulators (Mts-1, Mts-2, Lats) in MDA-MB231 cells transfected with vector, circYap or its linear precursor plasmid were examined by Western immunoblotting.

(e) Protein expression of eIF4A, eIF4B and eIF4E in MDA-MB231 cells transfected with vector, circYap or its linear precursor plasmid were examined by Western immunoblotting.
