# Supplementary material for: Translation of yes-associated protein (YAP) was antagonized by its circular RNA via suppressing the assembly of the translation initiation machinery
Source: Cell Death Differ. 2019 May 15;26(12):2758–73. doi: 10.1038/s41418-019-0337-2 (PMC7224378; doi:10.1038/s41418-019-0337-2)
Supplement: Supplementary file 7 — circYAP-Supplementary-Fig S5 [file 41418_2019_337_MOESM7_ESM.pdf]

a

Mut-1

gtcct**gaaggactacctacccttgttcggtac**tgtcaggatggagaaatttactatataaaccataagaacaagaccacctcttggctag
acccaaggcttgaccctcgttttgccatgaaccagagaatcagtcagagtgctccagtgaaacagccaccacccctggctccccagagcc
cacagggaggcgtcatgggtggcagcaactccaaccagcagcaacagatgcgactgcagcaactgcagatggagaaggagaggctgc
ggctg**tttgtcgttcttgtcg**atcggcag

Mut-2

g**acctcttcc**tgatggatgggaacaagccatgactcaggatggagaa**cggg**actatataaaccataagaacaagaccacctcttgg**gat**
gtggcaaggcttgaccctcgttttgccatgaaccagagaa**ag**tcagt**ct**gtgctccagtgaaacagccaccacccctggctccccagagc
ccacagggaggcgtcatgggtggca**cgttgagcaacc**agcagcaacagatgcgactgcagcaactgcagatgga**catc**gaga**cgga**g
cggctgaaacagcaagaactgcttcggcag

Mut-3

gtcctcttcctgatggatg**ccttgttc**ccatgactcaggatggagaaatttactatataaaccataagaacaagaccacctcttggctaga
cccaaggcttgaccctcgttttgccatgaaccagagaatcagtcagagtgctccagtgaaacagccaccacccctggctccccagagcc
cacagggaggcgtcatgggtggcagcaactccaaccagcagcaacagatgcgactgcagcaactgcagatggagaaggagaggctg
cggctgaaacagcaagaactgcttcggcag

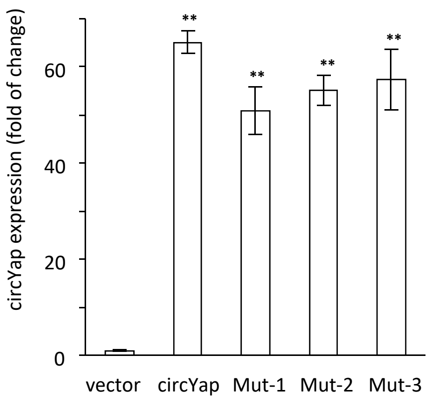

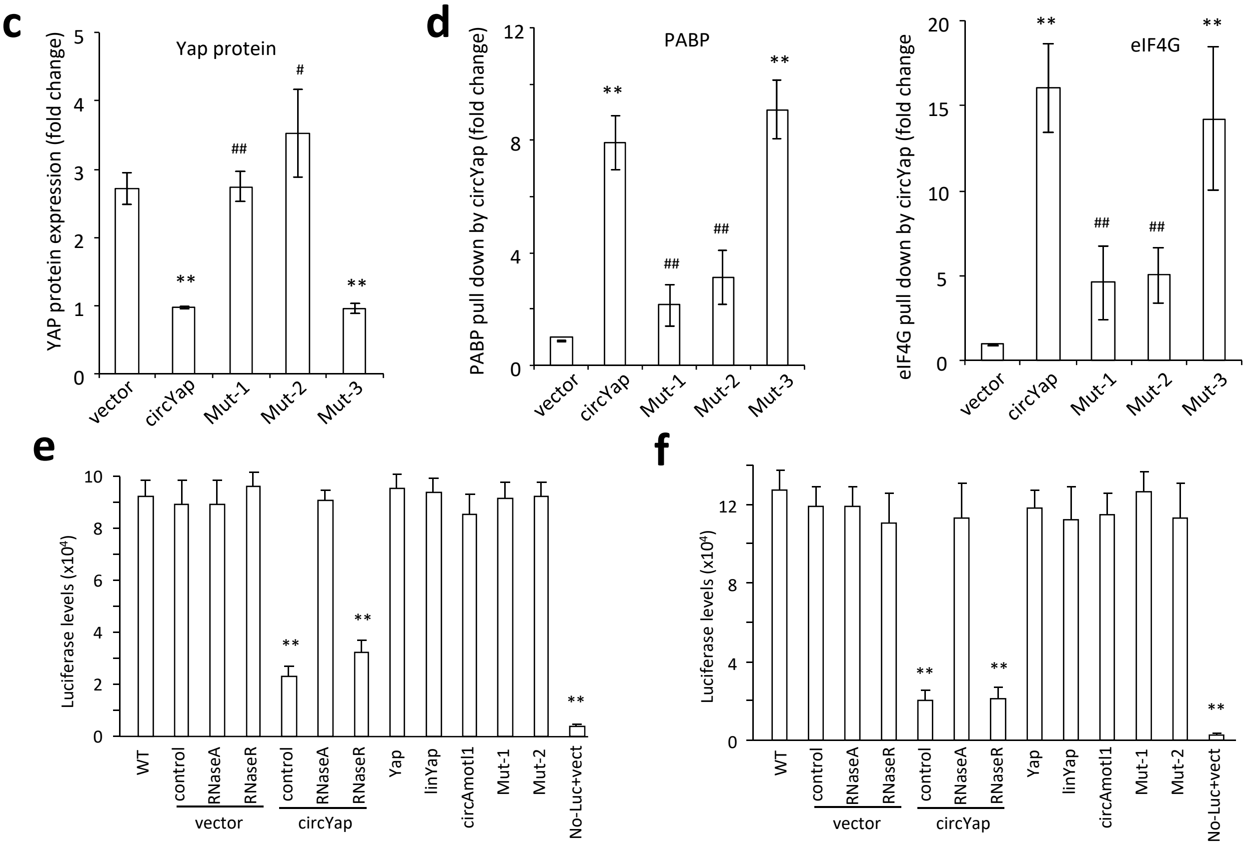

Supplementary Figure S5. Identify the binding sites of circYAP with YAP mRNA or PABP and eIF4G.

(a) The mutation was placed on the binding sites of circYAP with YAP mRNA (Mut-1) or with PABP and eIF4G (Mut-2) or some non-essential sites (Mut-3). The sequences of mutant inserts were listed.

(b) The circYap expression was examined by real-time PCR in MDA-MB231 cells stably transfected with vector, circYap, Mut-1, Mut-2 and Mut-3 plasmids. These results suggested the mutation of binding sites did not affect the formation of circYap.

(c) The densitometry of the blots in Figure 6e was analyzed with Quantity One program (Bio-Rad). The densities of the bands were normalized by GAPDH. n=3. **p<0.01 compared to vector control, $^{\#}$ p<0.05, $^{\#\#}$ p<0.01 compared to circYap.

(d) The PABP (left) and eIF4G (right) protein pulled down by circYap probe were examined by Western immunoblotting. The densitometry of the blots in Figure 6h was analyzed with Quantity One program (Bio-Rad). The densities of the bands were normalized by PABP or eIF4G input. n=3. **p<0.01 compared to vector control, ## p<0.01 compared to circYap.

(e-f) The specific translation inhibition of Yap mRNA by circYap was conducted in a cell-free extract of rabbit reticulocyte lysate. In vitro transcription was performed with a template of plasmid containing luciferaseT7luc(A) and Yap mRNA with 5'UTR (e) or both 5' and 3'UTR (f). Then, the capped luciferase RNA was supplemented with the RNA extracted from MDA-MB231 cells stably transfected with plasmid containing vector, circYap and its linear precursor (linYap), Yap mRNA, circAmotl1, or mutants of binding sites for in vitro translation. Among them, an equal amount of RNA extracts from vector and circYap group were also treated with RNase A or RNase R before in vitro translation. The luciferase activity was measured. n=3 **p<0.01 compared to wide type.
