# Supplementary material for: Translation of yes-associated protein (YAP) was antagonized by its circular RNA via suppressing the assembly of the translation initiation machinery
Source: Cell Death Differ. 2019 May 15;26(12):2758–73. doi: 10.1038/s41418-019-0337-2 (PMC7224378; doi:10.1038/s41418-019-0337-2)
Supplement: Supplementary file 8 — circYAP-Supplementary-Fig S6 [file 41418_2019_337_MOESM8_ESM.pdf]

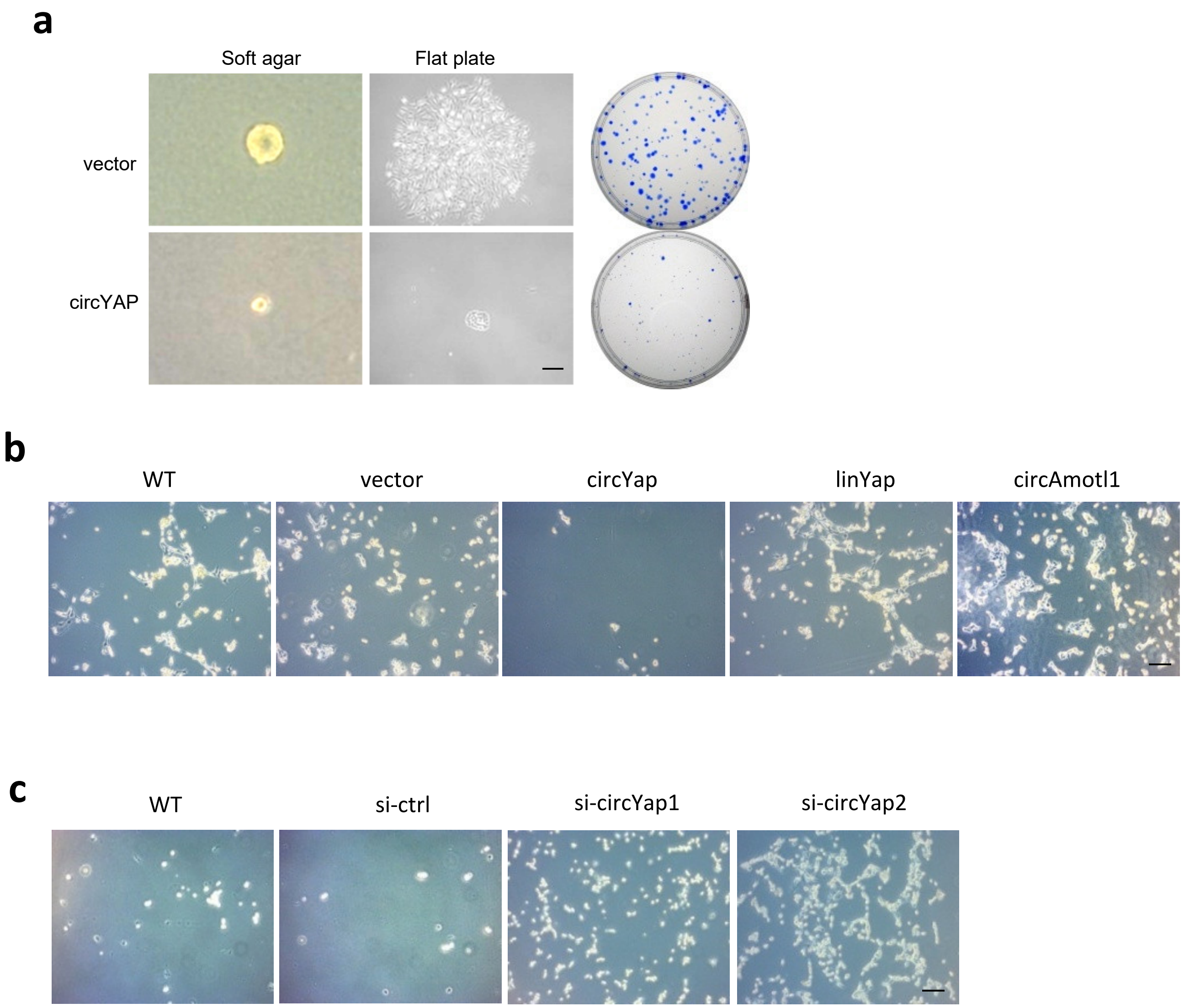

Supplementary Figure S6. Role of circYap in tumor cell colony formation and adhesion.
(a) The HepG2 cells transfected with circYap formed significantly less and smaller colonies than the vector transfected cells. Representative pictures are shown here. Scale bar, 200µm.
(b) MDA-MB231 cells were stably transfected with vector, circYap or its linear precursor (linYap), or angiomotin like protein-1 circRNA (circAmotl1) plasmids. Cells were inoculated in Petri dishes. Representative phase-contract pictures of the adhesive cells were taken 24 hours after inoculation. Scale bar, 100µm.
(c) MDA-MB231 cells were transfected with siRNA control (si-ctrl) or circYap siRNA (si-circYap1 and si-circYap2). Cells were inoculated in Petri dishes. Representative phase-contract pictures of the adhesive cells were taken 24 hours after inoculation. Scale bar, 100µm.
