# Supplementary material for: Translation of yes-associated protein (YAP) was antagonized by its circular RNA via suppressing the assembly of the translation initiation machinery
Source: Cell Death Differ. 2019 May 15;26(12):2758–73. doi: 10.1038/s41418-019-0337-2 (PMC7224378; doi:10.1038/s41418-019-0337-2)
Supplement: Supplementary file 9 — circYAP-Supplementary-Fig S7 [file 41418_2019_337_MOESM9_ESM.pdf]

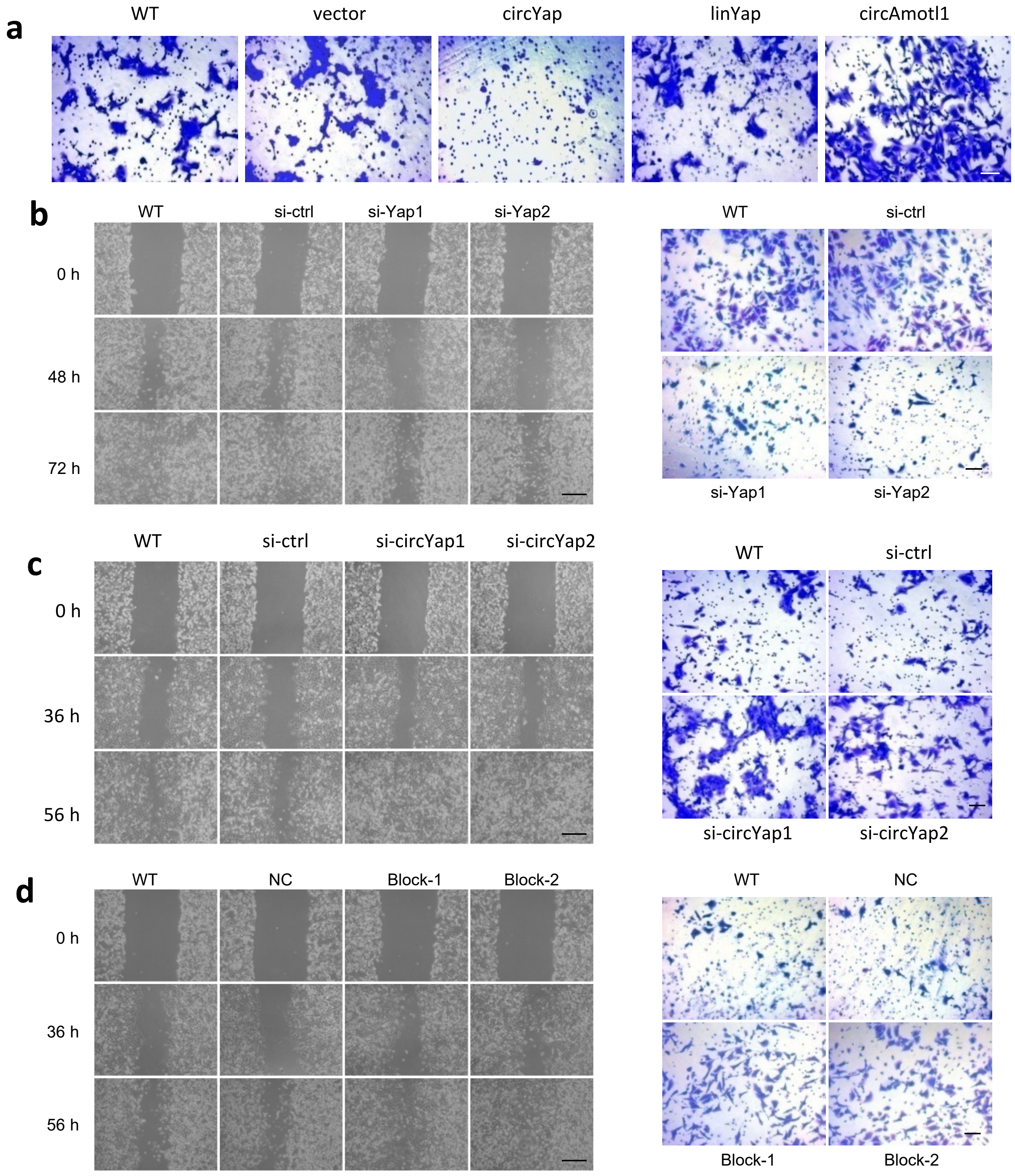

Supplementary Figure S7. Role of circYap in tumor cell invasion and migration.

(a) MDA-MB231 cells were stably transfected with vector, circYap or its linear precursor (linYap), or angiomotin like protein-1 circRNA (circAmotl1) plasmids. Cell invasion was determined by loading $1x10^5$ cells in transwells with 10% Matrigel in serum-free medium followed by incubation in the 24-well plates with 10% FBS containing medium. Representative phase-contract pictures of the cells were taken at 48 and 72 hours after cell inoculation. Scale bar, 200µm.

(b) MDA-MB231 cells were transfected with siRNA control (si-ctrl), Yap mRNA siRNA (si-Yap1 or si-Yap2). Left, the migration of the cells was examined with a scratch migration assay. Right, cell invasion was determined by loading $1x10^5$ cells in transwells with 10% Matrigel in serum-free medium followed by incubation in the 24-well plates with 10% FBS containing medium. Scale bar, 200µm.

(c) MDA-MB231 cells were transfected with siRNA control (si-ctrl), circYap siRNA (si-circYap1 or si-circYap2). Left, migration of the cells was examined with a scratch migration assay. Right, cell invasion was determined by loading $1x10^5$ cells in transwells with 10% Matrigel in serum-free medium followed by incubation in the 24-well plates with 10% FBS containing medium. Scale bar, 200µm.

(d) MDA-MB231 cells were transfected with negative control (NC), blocking oligos (block-1 or block-2). Left, cell migration was examined with a scratch migration assay. Right, cell invasion was determined by loading $1x10^5$ cells in transwells with 10% Matrigel in serum-free medium followed by incubation in the 24-well plates with 10% FBS-containing medium. Scale bar, 200µm.
