# Supplementary material for: Translation of yes-associated protein (YAP) was antagonized by its circular RNA via suppressing the assembly of the translation initiation machinery
Source: Cell Death Differ. 2019 May 15;26(12):2758–73. doi: 10.1038/s41418-019-0337-2 (PMC7224378; doi:10.1038/s41418-019-0337-2)
Supplement: Supplementary file 6 — circYAP-Supplementary-Fig S4 [file 41418_2019_337_MOESM6_ESM.pdf]

a

Binding site 1
query YAP (5396 nts) vs. target circYAP (296 nts)
>circYAP
>YAP
.((..(.((((((((((((......((.....((((((((.&.)))))))......))....))))))..))))))).)).
256,295 : 63,101 (-30.60)

Binding site 2
query circYAP (296 nts) vs. target YAP (5396 nts)
>YAP
>circYAP
.((((((((..((.(.((((((((((((.....(((.((.&))))))))))))).....)))).).))...)).)))))))
352,392 : 1,40 (-30.70)

b

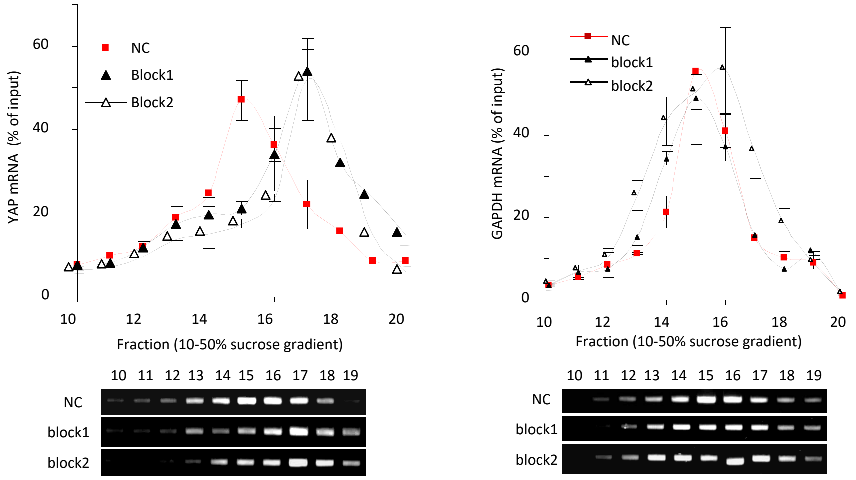

c

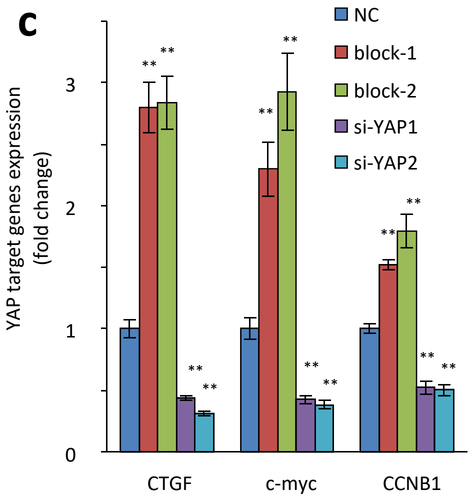

d

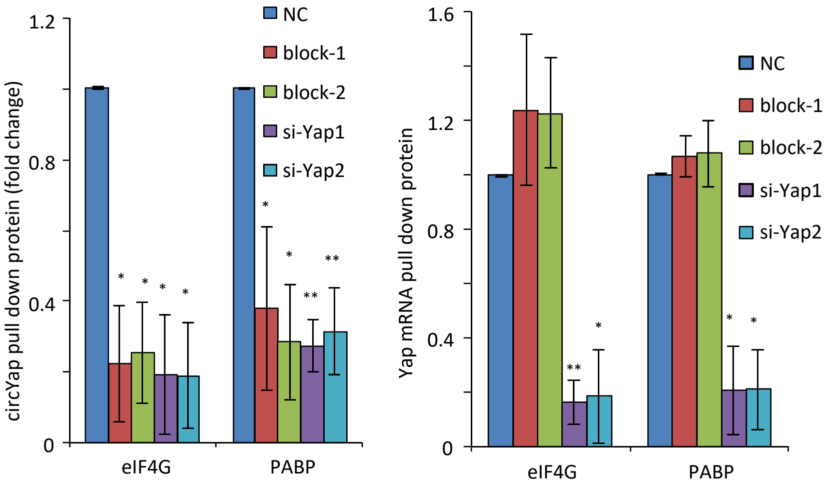

e

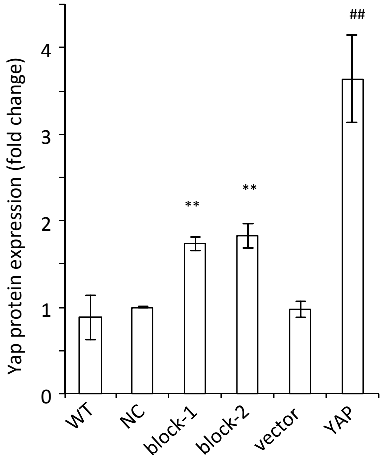

Supplementary Figure S4. Identify the binding sites of circYAP with YAP mRNA.
(a) The binding sites of circYAP with YAP mRNA were identified by using the RNAplex software.
(b) Polysome from of MDA-MB231 cells transfected with the negtive control (NC) or blocking oligos (block-1 and block-2) were extracted and subjected to 10% to 50% sucrose gradient by ultracentrifuge. Twenty-four polysome fractions were collected from top to bottom followed by RNA extraction. Yap (right) and GAPDH (left) mRNA expression in each fraction were determined by real-time PCR (upper) and visualized by DNA agarose gel (lower). n=3.
(c) The expression of Yap protein target genes (CTGF, c-myc and CCNB1) was examined by real-time PCR in MDA-MB231 cells transfected with negative control (NC), blocking oligos (block-1 and block-2), and siRNAs for Yap mRNA (si-Yap1 and si-Yap2). n=4. $^{**}p<0.01$ compared to negative control. These results suggested the transcription of Yap target genes could be upregulated due to blocking the binding of circYap and Yap mRNA increased Yap protein translation.
(d) The densitometry of the blots in Figure 5c was analyzed with Quantity One program (Bio-Rad). The density of the bands was normalized by PABP or eIF4G input. The left panel showed the PABP and eIF4G protein pulled down by circYap probe. The right panel showed the PABP and eIF4G protein pulled down by Yap mRNA probe. n=3. $^{*}p<0.05$, $^{**}p<0.01$ compared to vector control.
(e) The densitometry of the blots in Figure 5e was analyzed with Quantity One program (Bio-Rad). The densities of the bands were normalized by GAPDH. n=3. $^{**}p<0.01$ compared to negative control, $^{\#\#}$ $p<0.01$ compared to vector.
